# Study protocol: a pragmatic, stepped-wedge trial of tailored support for implementing social determinants of health documentation/action in community health centers, with realist evaluation

**DOI:** 10.1186/s13012-019-0855-9

**Published:** 2019-01-28

**Authors:** Rachel Gold, Arwen Bunce, Erika Cottrell, Miguel Marino, Mary Middendorf, Stuart Cowburn, Dagan Wright, Ned Mossman, Katie Dambrun, Byron J. Powell, Inga Gruß, Laura Gottlieb, Marla Dearing, Jason Scott, Nadia Yosuf, Molly Krancari

**Affiliations:** 1 Kaiser Permanente–Center for Health Research, 3800 N. Interstate Ave., Portland, OR 97227 USA; 2 OCHIN, Inc, 1881 SW Naito Pkwy, Portland, OR 97201 USA; 3 Oregon Health and Science University, 3181 S.W. Sam Jackson Park Rd., Portland, OR 97239 USA; 4 University of North Carolina at Chapel Hill, 1105C McGavran-Greenberg Hall, Chapel Hill, NC 27599-7411 USA; 5 University of California, San Francisco, 500 Parnassus Avenue, San Francisco, CA 94143 USA

**Keywords:** Social determinants of health, Electronic health records, Community health centers, Implementation, Implementation strategies

## Abstract

**Background:**

National leaders recommend documenting social determinants of health and actions taken to address social determinants of health in electronic health records, and a growing body of evidence suggests the health benefits of doing so. However, little evidence exists to guide implementation of social determinants of health documentation/action.

**Methods:**

This paper describes a 5-year, mixed-methods, stepped-wedge trial with realist evaluation, designed to test the impact of providing 30 community health centers with step-by-step guidance on implementing electronic health record-based social determinants of health documentation. This guidance will entail 6 months of tailored support from an interdisciplinary team, including training and technical assistance. We will report on tailored support provided at each of five implementation steps; impact of tailored implementation support; a method for tracking such tailoring; and context-specific pathways through which these tailored strategies effect change. We will track the competencies and resources needed to support the study clinics’ implementation efforts.

**Discussion:**

Results will inform how to tailor implementation strategies to meet local needs in real-world practice settings. Secondary analyses will assess impacts of social determinants of health documentation and referral-making on diabetes outcomes. By learning whether and how scalable, tailored implementation strategies help community health centers adopt social determinants of health documentation and action, this study will yield timely guidance to primary care providers. We are not aware of previous studies exploring implementation strategies that support adoption of social determinants of action using electronic health and interventions, despite the pressing need for such guidance.

**Trial registration:**

clinicaltrials.gov, NCT03607617, registration date: 7/31/2018—retrospectively registered

**Electronic supplementary material:**

The online version of this article (10.1186/s13012-019-0855-9) contains supplementary material, which is available to authorized users.

## Background

“Social determinants of health” impact health risks and outcomes [1–12]. For example, adverse social determinants (e.g., chronic stress, poverty, lack of access to healthy foods/safe exercise) create barriers to acting on diabetes care recommendations, increasing risks of poor diabetes outcomes [13–21]. Through such mechanisms, social determinants contribute to health disparities, hamper efforts to implement guideline-based care, and break the link between care quality and health outcomes [3, 22–25]. A small but growing body of research shows that documenting patients’ social determinants of health in healthcare settings leads to improved receipt of social services and improved health outcomes [26–29]. Social determinants documentation in electronic health records can also improve care teams’ ability to track and respond to patients’ social needs systematically [30–33].

Thus, numerous national leaders now recommend documenting social determinants of health in electronic health records, and taking action to address social determinants of health (e.g., referring patients to social service agencies; adapting care plans as needed) [31, 34–40]. Since such documentation/action may become required for some care providers, especially those in Accountable Care Organizations [41], many health care delivery systems are exploring ways to incorporate social determinants of health screening/action into routine care [28, 42–44], including through more routine documentation of patient-reported social determinants of health in electronic health records [32, 33, 45, 46].

Systematic electronic health record documentation of patients’ social determinants of health needs could help care teams understand potential impacts on their patients’ health and ability to act on care recommendations [28, 40, 47–51] and adjust care plans accordingly (e.g., prescribe medications that do not require refrigeration if a patient is homeless) [29, 52], or intervene to address social determinants of health (e.g., through referrals to community resources) [29, 52–57]. Well-documented social determinants of health could also identify needed social service resources [47] and inform health care payment structures that account for the social vulnerability of a clinic’s patient population [58–60]. And, while emergent research suggests the health benefits of social determinants documentation/action, improving such documentation in electronic health records will enable further scientific assessment of which social determinants most impact specific patients’ health, and how clinical teams can intervene to address these impacts.

These benefits cannot accrue without effective strategies for implementing social determinants of health data documentation/action, but little evidence yet exists to guide integrating social determinants of health documentation into standard practice [28, 42, 61–64]. The need for such guidance is especially urgent in primary care community health centers, which serve patients whose health risks are high, and whose exposure to social determinants of health are profound [17, 20, 21, 65–67]. Although community health centers have long sought to understand and address the social factors that impact health, their efforts have typically been ad hoc and rarely documented in electronic health records [15, 24, 25, 65, 68–75].

Some efforts to help community health centers and other primary care settings adopt systematic social determinants of health documentation in electronic health records are underway. The National Association of Community Health Centers’ “Protocol for Responding to and Assessing Patient Assets, Risks, and Experiences” (PRAPARE) [76] outlines how community health centers can collect patient-reported social determinants of health data and suggests electronic health record-based social determinants of health data documentation tools.

Our team built on PRAPARE in a recent pilot study (R18DK105463) that sought to optimize the documentation and presentation of social determinants of health data within standard electronic health record functions. (We believe this was the first US study on documenting standardized social determinants of health data using electronic health record-based tools in community health centers) [45, 46]. We developed a suite of electronic health record-based social determinants of health data tools [45, 77] and activated them in a network of > 500 community health centers with a shared electronic health record in June 2016. These tools are described elsewhere [46, 77]. Three pilot study clinics were also given electronic health record tools to facilitate referring patients with social determinants of health needs to community resources. These tools enable staff to give patients information about local services; provide “internal” referrals to social workers, community health workers, etc.; and help patients make appointments with those services. The tools’ lists of available community resources must be manually updated by clinic staff.

Our pilot study demonstrated the feasibility of developing electronic health record tools for social determinants of health documentation/action. It also revealed myriad implementation barriers. Some barriers were similar to those associated with implementing other patient-reported data collection [78–84], such as difficulties with optimizing workflows/minimizing logistical burdens; staff turnover; adequately training relevant staff; billing for staff time spent collecting and acting on these data; knowing which patient-reported measures are most important; having resources for addressing identified needs; and ensuring that the right staff see the needed data at the right workflow step and can respond to these data [78, 79, 81–90]. Barriers specific to adoption of social determinants of health documentation/action included the need to change perceptions of healthcare teams’ responsibilities; lack of clarity about how to make social determinants of health-related “referrals”; clinic staff concerns about collecting data on social determinants of health needs when no “action” could be taken to address those needs; limited knowledge of how to use the electronic health record for this purpose; false-positive screening results (e.g., patient has food insecurity, but already accesses a food bank); the initial lack of a method for documenting whether patients want help; and inadequate infrastructure, incentives, and decision support for effective social determinants of health screening/action.

Such barriers could substantially hamper implementation of social determinants of health documentation, thus impeding community health centers’ (and others’) ability to use social determinants of health data. The “ASCEND” trial (1R18DK114701-01, ApproacheS to Community Health Center ImplEmeNtation of Social Determinants of Health Data Collection and Action), described here, will test whether and how providing tailored, scalable, pragmatic implementation support helps community health centers adopt social determinants of health screening documentation/action using electronic health record tools. To our knowledge, no previous trials have formally tested implementation strategies targeting electronic health record-based social determinants of health documentation/action [91]. Secondary analyses will assess impacts of social determinants of health documentation and action on care quality and biomarkers in patients with/at risk for diabetes (an expected subset of screened patients); only a few previous studies have assessed such impacts [26–28, 57]. Study results could inform diverse national efforts to increase social determinants of health documentation and action.

This study will directly address dissemination and implementation science priorities by evaluating the impact of providing tailored implementation strategies [92–96], and demonstrating a method for tracking such tailoring [97–100]. Through this method, we will report on how support was tailored at each implementation step. To augment this information, our realist evaluation will identify context-specific pathways through which these tailored strategies effect change [101, 102]. This study was approved by the Kaiser Permanente Northwest Institutional Review Board.

## Methods

This 5-year study began in September 2017. It is being conducted at OCHIN (not an acronym), a non-profit health center-controlled network that hosts and centrally manages an Epic© electronic health record for > 500 primary care community health centers located in 18 states, as of July 2018 [103–105]. OCHIN’s electronic health record is shared by its member community clinics, making it the nation’s largest community health center network on a single electronic health record instance. Table 1 shows the characteristics of OCHIN community health centers’ patients seen between June 2016 and May 2018. The table shows that OCHIN community health centers’ patients’ socioeconomic risks are reflected in social determinants of health data already collected per federal requirements: 21% are uninsured and 63% are publicly insured; only 37% are white; 31% are of Hispanic ethnicity, 28% primarily non-English speakers, and 62% are from households < 138% of the federal poverty level.

**Table 1:** Demographic characteristics of OCHIN patients with an ambulatory visit/office encounter, 6/24/2016–5/17/2018

	*N*	%
Total	1,739,812	100
Sex		
Female	972,929	55.9
Male	766,733	44.1
No information	150	0.0
Race/ethnicity		
Hispanic	545,346	31.3
Missing	100,472	5.8
Non-Hispanic Black	298,095	17.1
Non-Hispanic other	158,067	9.1
Non-Hispanic White	637,832	36.7
Language		
English	1,234,791	71.0
Spanish	353,289	20.3
Other	123,515	7.1
No information	28,217	1.6
Age at first encounter in study period		
0–9	259,146	14.9
10–19	247,443	14.2
20–29	282,537	16.2
30–39	269,013	15.5
40–49	220,378	12.7
50–59	225,785	13.0
60–69	154,131	8.9
> = 70	81,377	4.7
No information	2	0.0
Household income as % of federal poverty level		
<= 138%	1,078,603	62.0
> 138%	265,644	15.3
No information	395,565	22.7
Current insurance status		
Medicaid	897,582	51.6
Medicare	156,719	9.0
Other public	42,906	2.5
Private	270,515	15.5
Uninsured	372,090	21.4

**Fig. 1:**
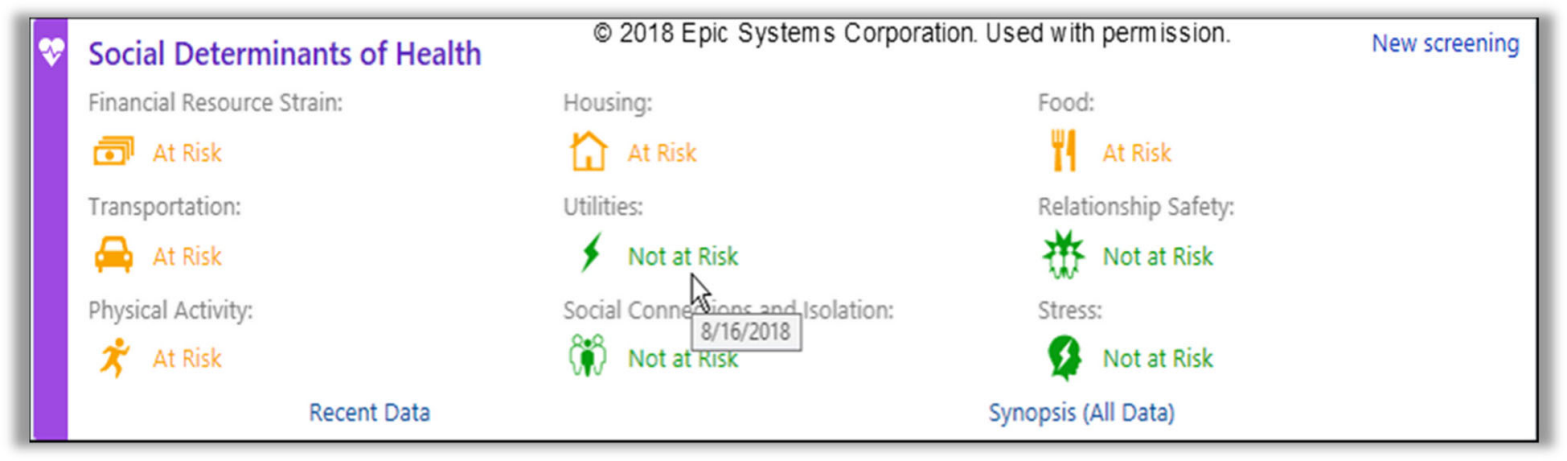
Social determinants of health summary view

The social determinants of health data tools in OCHIN’s electronic health record are the “innovation” whose adoption is targeted in this study. The tools were fine-tuned for the current study, based on lessons from the pilot study and formative analyses (described below), and to ensure their alignment with the Epic© electronic health record’s 2018 social determinants of health module. They include options for clinic staff to document social determinants of health data directly into the electronic health record, or for patients to do so through the patient portal or a tablet at the clinic. If patients complete social determinants of health screenings on paper, the data must be entered into the electronic health record by clinic staff. Social determinants of health screening results and past social determinants of health-related referrals are shown in an social determinants of health summary, with positive screening results highlighted visually (Fig. 1). The social determinants of health questions in the tools align with those recommended by several national groups [30, 45, 76, 77].

We will provide step-by-step tailored implementation support to the 30 study community health centers (details below) and evaluate how effectively this intervention supports such clinics’ adoption of social determinants of health screening/action, as documented in the electronic health record. This is a mixed-methods, pragmatic, stepped-wedge, cluster-randomized trial, with a hybrid type 3 implementation-effectiveness design: we focus on adoption of electronic health record documentation of social determinants of health data and processes, and also consider the health impacts of this adoption [106–109]. Primary outcomes are adoption of electronic health record-based social determinants of health documentation/action; secondary outcomes are the impact of such adoption on the health of adults with/at risk for diabetes. (Study clinics will decide which patients they want to screen; we will conduct secondary analyses among those with diabetes.) Cluster randomization enables controlling for clinic-level characteristics, appropriate to our primary outcomes of clinic-level changes. The stepped-wedge design, with six wedges, enables us to provide the intervention to five community health centers at a time while ensuring that all study clinics eventually receive the intervention, which will help with participation and retention and has advantages over parallel cluster-randomized trials in terms of statistical power [110] (Table 2).

**Table 2:** Stepped-wedge design

Study step	Study months								
	1–6	7–12	13–18	19–24	25–30	31–36	37–42	43–48	49–54
Wedge 1 (CHCs 1–5)	C	C, BL	I	FU	FU	FU	FU	FU	FU
Wedge 2 (CHCs 6–10)	C	C	C, BL	I	FU	FU	FU	FU	FU
Wedge 3 (CHCs 11–15)	C	C	C	C, BL	I	FU	FU	FU	FU
Wedge 4 (CHCs 16–20)	C	C	C	C	C, BL	I	FU	FU	FU
Wedge 5 (CHCs 21–25)	C	C	C	C	C	C, BL	I	FU	FU
Wedge 6 (CHCs 26–30)	C	C	C	C	C	C	C, BL	I	FU

*C*, control period; *I*, intervention period; *BL*, baseline survey; *FU*, follow-up

### Conceptual guide

This study is guided by the “building blocks of primary care” [111], which outlines components essential to high-performing primary care practices, building on Starfield’s four pillars of primary care practice, and elements of the Joint Principles and Primary Care Medical Home recognition standards [112]. Its foundational “building blocks” are (1.) engaged leadership; (2.) data-driven improvement using electronic health records; (3.) empanelment; (4.) team-based care. The intervention directly addresses these four building blocks as they relate to social determinants of health screening/action using electronic health record tools (Tables 3 and 4). A realist evaluation framework will guide our evaluation of the causal processes that lead to intervention outcomes [113]. Measurement of implementation success is guided by the RE-AIM framework [114]. Analysis details are given below.

**Table 3:** Characteristics of study implementation strategies, per Proctor et al. [116]

Implementation strategies	Proctor et al. categories for reporting on implementation strategies					
	Target	Actor	Action	Outcome	Temporality	Dose
1. The clinic action plan (CAP)	SDH operational/clinical champions	Implementation support team (IST)	Providing the strategy/materials/training to the clinic	Champion understands and tracks needed tasks	At the beginning of wedge	Once
2. Technical assistance—implementing SDH screening				Champion has needed tools to make decisions about SDH implementation (workflows; rollout plans)	When clinic is ready to develop workflow and rollout plans	Once, although revisited as needed
3. Technical assistance—using the EHR				Champion has the information needed to train clinic staff in the EHR’s SDH tools	When the champion is ready to train staff on the tools	Once, although revisited as needed
4. Ongoing technical assistance, tailored problem-solving						
4a. Bi-monthly hour-long webinars/office hours/peer support				Champions learn about each implementation step and seek expert/peer advice related to that step	Throughout the 6-month intervention	Twice monthly
4b. Monthly hour-long coaching call				Champions receive one-on-one coaching to address their clinic’s needs	Throughout the 6-month intervention	Once monthly
4c. Email questions				All clinics’ champions receive information about implementation processes and problem-solving at all wedge sites	Throughout the 6-month intervention	Once monthly

**Table 4:** Implementation support components

CAP step	Tasks for this step	Format of support	Specifics of implementation support strategy	Implementation strategy type, per ERIC categorization scheme [128]
Step 1. Create an “SDH team.”	Obtain leadership support (BBPC #1). Identify, orient clinic champion/study contact.	Materials	For clinic leaders: benefits of SDH documentation/action; leaders’ role in supporting SDH process adoption	Recruit, designate, train for leadership; orientation materials
			Clinician champion orientation, task summary materials	
			Project champion orientation, task \summary materials	
			Draft email from leadership to clinic staff alerting staff to SDH plan	Technical assistance
		Office hours	Covering: (1) orienting champions; (2) goal setting	Identify/prepare champions; recruit, designate, train for leadership; orientation materials; peer-to-peer learning
		Call	With implementation support team: orientation	Technical assistance
Step 2. Identify clinic goals.	Identify clinic’s goals for SDH screening, and which patients will be screened for which SDH measures.	Materials	Decision tools: why do you want to collect SDH data? What do you hope to accomplish? What do you plan to do with the SDH data? Which patients do you want to screen? How often? For which SDH?	Goal identification/implementation blueprint
			Written recommendations/key considerations for selecting clinic goals	Goal identification; technical assistance
			Summary of the clinic’s stated goals	Goal identification
		Office hours	Covering: (1) goal setting; (2) learning the EHR tools	Goal identification/implementation blueprint; peer-to-peer learning
		Call	With implementation support team: identify goals	Goal identification/implementation blueprint
Step 3. Create an “SDH plan.”	Create a workflow plan for SDH documentation, and (if desired) SDH data review and action (BBPC #3, 4). Create a rollout plan.	Materials	Planning tools: SDH documentation workflow; SDH data review/action workflow; workflow implementation rollout	Technical assistance
			Resource list (PRAPARE, HealthLeads, etc.)	
			Guides to using EHR’s SDH data tools: in workflows; in SDH documentation, on site or via patient portal; to review SDH; for SDH referral-making (with guidance on creating a social service resource list)	
			Written materials: pros and cons of different SDH documentation workflow options; key considerations based on other CHCs’ experience	
			Summary of the clinic’s stated workflow plan	Goal identification/implementation blueprint; technical assistance
		Call	With implementation support team: workflow development, use of workflow planning tools, rollout plan	Technical assistance
		Office hours	Covering: (1) workflow planning; (2) EHR tools within workflows	Peer-to-peer learning; technical assistance
Step 4. Train clinic staff in the “SDH plan.”	Orient staff: staff meeting. If SDH plan changes, orient staff. Train new staff as needed.	Materials	Orientation webinar for clinic staff; review clinic’s goals and workflow plan; include staff discussion of potential barriers/how to address them.	Educational meeting/materials; goal identification
			Written materials: how to orient clinic staff to SDH documentation and action, based on other CHCs’ experiences	Educational meeting
			Template slides/handouts for updating staff and/or training new staff	Educational meeting; technical support
		Call	With implementation support team: how to train staff	
		Office hours	Covering: (1) how to train staff; (2) how to create target population reports and adoption reports	Peer-to-peer learning; technical assistance
Step 5. Roll out the “SDH plan.”	Review adoption rates on a regular basis (BBPC #2). Iterate/revise rollout, workflows as needed.	Materials	Guides: using SDH data tools to review SDH documentation/action data; using SDH documentation data to track progress; testing workflows	Audit and feedback
		Call	With implementation support team: develop strategy for testing workflows, addressing barriers, rollout, review of adoption progress; how to track SDH adoption progress using data tools; how to revise workflows, rollout plan as needed	Audit and feedback; technical assistance; practice facilitation/small tests of change; tailor strategies
		Tools	Provide monthly adoption reports	Audit and feedback; tools for quality monitoring
		Office hours	Covering: how to iterate and refine workflows; other topics identified as needed by the clinics or the IST	Peer-to-peer learning; technical assistance; ongoing consultation

*Abbreviations*: *SDH* social determinants of health; *IST* implementation support team; *Q&A* questions and answers; *BBPC* building blocks of primary care

### Recruitment and randomization

We recruited eight community health center organizations from OCHIN’s membership for formative interviews with clinic staff, targeting clinics with prior social determinants of health documentation in the electronic health record’s social determinants of health data tools. Thirty additional OCHIN member community health centers will be recruited in two waves for the trial portion of the study, targeting those who want to initiate or improve their social determinants of health documentation/action efforts. The first wave of 15 practices was recruited in the spring of 2018 and block-randomized to wedges 1–3 with 5 clinics per wedge. The second wave of 15 practices will be recruited in 2019 and randomized to wedges 4–6 as in wave 1. This two-wave process ensures that no recruited clinics will wait more than a year to receive the intervention, important both for recruitment and because the rapidly changing social determinants of health screening landscape means clinics’ needs and interests may change between recruitment periods. As our primary outcomes can be derived historically from the electronic health record, we will obtain pre-intervention data at all time points as required to evaluate stepped-wedge trials. All study clinics will receive the same intervention; randomization staggers the timing of when the intervention starts (Table 2).

Study clinics will be asked to identify a clinician champion and/or a “Social determinants of health Operational Champion” to oversee the clinic’s social determinants of health implementation efforts, and take part in the intervention’s implementation support activities. The clinics will receive a description of the tasks involved with each role and may select staff for these roles as they deem appropriate.

### The implementation support intervention

Implementation support will be provided to one “wedge” of five community health centers at a time by a multi-disciplinary implementation support team, for 6 months per wedge (Tables 3 and 4). Implementation support team members have expertise in social determinants of health, clinic workflows and practice change implementation, and electronic health record use. (If any needed competencies are identified that the implementation support team does not have, we will bring in the needed expertise and document the skills needed to support community health centers’ social determinants of health screening/action adoption.) We expect that implementation support team members will spend approximately 1 h/month in calls with each study clinic, 2 h/month on office hours, 1–2 h/week to discuss the clinics’ progress and needs internally, and 1–2 h/week to respond to clinic emails, for a total of 15–23 h per month to support five clinics. We will document whether more or less time is needed.

The tailored support uses implementation strategies selected for their demonstrated effectiveness at supporting practice change [98, 115–126], results from our pilot study, and potential scalability. They include staff training, technical assistance, audit and feedback, goal identification, leadership engagement, practice coaching, peer-to-peer learning, orientation materials, and implementation guides. This approach is based on evidence that practice change is best supported by a combination of implementation strategies, e.g., “change toolkits” are more likely to be adopted if guidance for their use is also provided. Table 3 shows characteristics of each implementation strategy, as per Proctor et al.’s implementation strategy reporting recommendations [117].

In each implementation step, these strategies will be supported by specific materials and interactions with the study clinics (Table 4). This “lesson plan” approach, in which the implementation support team provides each clinic with just the materials needed for their next implementation step (although all materials will be available on a learning management system), is designed to avoid overwhelming the clinics with too much information at once [127].

#### Implementation strategies 1–4

**Fig. 2:**
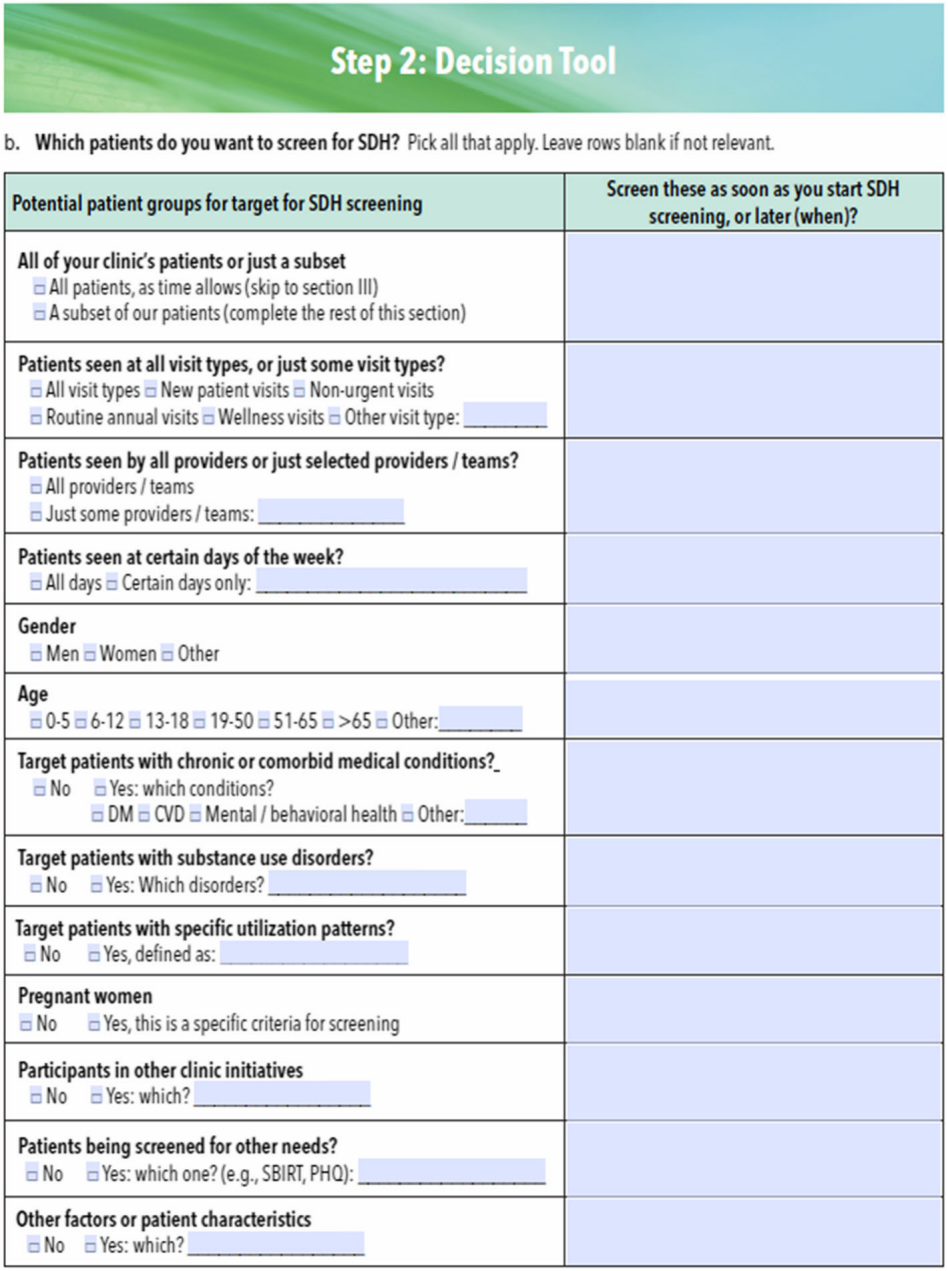
Decision tool: which patients does the clinic want to target for social determinants of health screening?

**Fig. 3:**
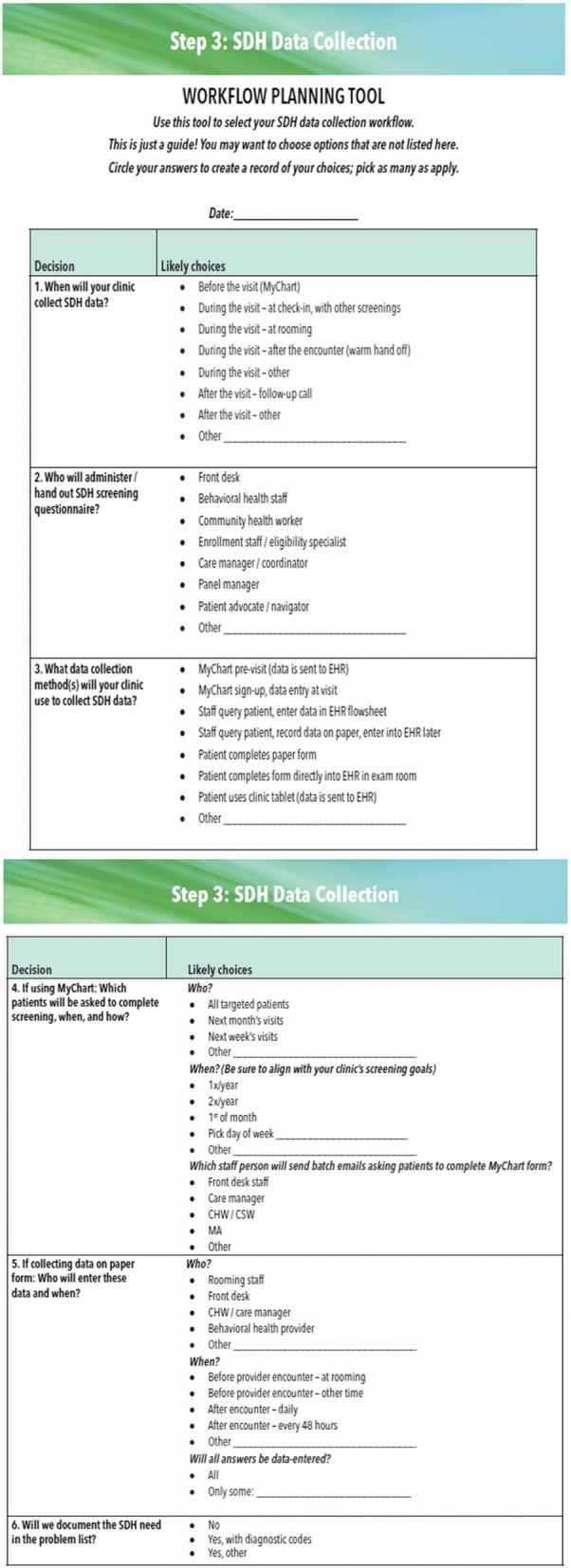
Social determinants of health workflow planning tool

1. *The clinic action plan*. This step-by-step guide to implementing social determinants of health data documentation/action (first two columns, Table 4) was developed based on findings from our pilot study.
2. *Technical assistance—implementing social determinants of health screening.* We will provide written materials to support each clinic action plan step. These were informed by social determinants of health implementation guides developed by national groups (e.g., PRAPARE, HealthLeads) [76, 128] with input from these groups and by learnings from our pilot study [46, 77]. The materials include recommendations and decision tools for each step. Figures 2 and 3 are examples of these decision tools. The entire implementation guide is in Additional file 1.
3. *Technical assistance—using the electronic health record.* We found no existing social determinants of health implementation guides that emphasize use of electronic health record data tools. Our team developed training materials on the use of the electronic health record’s tools for social determinants of health documentation/action. They include tips on using the social determinants of health tools in workflows; illustrated guides to using the electronic health record tools for social determinants of health screening/action and for monitoring the clinic’s tool use adoption; and information on how to identify community social service agencies to which patients can be referred.
4. *Ongoing technical assistance, tailored problem-solving:*
  - *Bi-monthly hour-long webinars/office hours/peer support:* The implementation support team will hold “office hours” via webinar every 2 weeks; study clinics will be encouraged to attend and submit questions in advance. Each webinar will focus on one aspect of social determinants of health adoption, determined by clinic request/the coach’s knowledge of the clinics’ progress. To support peer-to-peer learning, we will ask clinics that have made progress in a given step to present on their success, and encourage discussion across sites.
  - *Monthly hour-long coaching call:* A member of the implementation support team will meet with each clinic’s champion by phone to review the clinic’s progress, ask about barriers/facilitators to social determinants of health documentation/action implementation, and help as needed.
  - *Email questions:* Study clinics will be encouraged to email the implementation support team with questions; the implementation support team will respond within two workdays. Content from these emails and the monthly webinars will be summarized and shared with all clinics in a given wedge via a monthly email.

### Tailoring

A growing body of research [97–99] supports tailoring implementation support to meet local needs, i.e., customizing implementation support rather than providing a one-size-fits-all strategy. We will tailor implementation support to each clinic’s specific needs and track this customization. The implementation support team will first review each clinic’s baseline data, consider what might address each clinic’s needs, and tailor the implementation support plan as feasible. For example, if a given clinic does not have experience using their own data to drive improvement efforts, the implementation support team will plan to offer additional training on how to do so. During the intervention period, clinics will complete a bi-monthly web-based survey describing their progress. We will track which support strategies the clinics needed at each clinic action plan step, and if a clinic is stuck at a certain step, the implementation support team will identify additional implementation support that might help. For example, if a clinic gets stuck on step 2 after receiving the support listed in Table 4, we might provide additional calls, trainings, or materials to help with specific encountered barriers. We will document the precise implementation steps where study clinics faced barriers, the support provided to address those barriers, and whether that support helped. Thus, the clinic action plan is a pragmatic tool for guiding and tracking the provision of tailored implementation support.

### Data collection and analysis

#### Formative data collection and analysis (year 1; completed)

At time of writing, we are at the start of study year 2. In study year 1, we measured social determinants of health data collection among all OCHIN community health centers, using extracted electronic health record data. We recruited eight community health center organizations with high social determinants of health documentation rates (as identified in these formative analyses) to take part in exploratory semi-structured interviews. Clinics were asked to identify six staff members who played different roles related to social determinants of health documentation. The interviews explored barriers/facilitators to electronic health record-based social determinants of health data collection/use, and experiences with the electronic health record’s social determinants of health data tools. Results were used to identify needed improvements to the social determinants of health data tools and informed development of the implementation support intervention. Formative data analysis results will be reported in a future publication.

#### Implementation data collection and analysis (years 2–5)

##### Quantitative evaluation

All quantitative data will be extracted from study clinics’ shared electronic health record. Outcome measures are guided by the RE-AIM framework [114] (Table 5). Outcomes will be measured monthly in all study clinics at every period. Each wedge provides data points in both control and intervention conditions.

**Table 5:** Study outcomes

RE-AIM component	Outcome measures
Reach	• Rate of clinic encounters where SDH is documented: among any patients and targeted patients
Effectiveness—secondary outcome (among patients with/at risk for diabetes, a subset of study CHC patients)	Rate of all/targeted patients seen that month who have: • Controlled diabetes risks (BP < 140/80; A1c < 7.0%; BMI < 30; LDL < 100 mg/dL) • Incident comorbidities, e.g., retinopathy, neuropathy • Up-to-date diabetes tests (lipid panel, HbA1c, eye/foot exams)
Adoption—primary outcome	Rate of • All patients seen for whom SDH data are documented • Targeted patients seen for whom SDH data are documented • Patients with an identified SDH need with documented referrals to community agencies: overall; among those who requested clinic assistance; and according to reported SDH needs
Implementation	• Participation in implementation support activities • Realist evaluation results
Maintenance	• All measures over time • Change in diabetes risks/measures over time

To compare the effect of the intervention with usual practice on social determinants of health outcome measures in a stepped-wedge design, we will utilize generalized linear mixed models with random effects for clinic. Random effects for state will be considered to account for clustering of practices within states. This model will incorporate independent variables, take into account the general time trend, and allow for the intervention effect to grow over time. We will estimate the intervention effect with the within-site difference between social determinants of health collection rates pre- and post-intervention, averaging across practices and accounting for possible secular trends which might confound results. As our statistical tests are specified a priori and our proposed social determinants of health outcome measures are highly related, we will report *p* values rather than adjust for multiple comparisons [129, 130]. If significant differences in key clinic characteristics between wedges remain post-randomization, we will use propensity score methods to reduce observed bias and thereby minimize external threats to validity [131, 132].

In secondary analyses focused on a diabetes population, we will measure intervention-associated changes in clinical measures reflecting diabetes risk management (blood pressure, hemoglobin A1c, body mass index, lipids, etc.), rates of incident comorbidities, and rates of patients up-to-date on key diabetes tests (lipid panel annually, hemoglobin A1c within 6 months, eye/foot exams). We hypothesize that patients at intervention clinics for whom social determinants of health data are collected will have significant improvements in these measures by the end of the study period, compared to those at control clinics. Data for these analyses will be extracted from OCHIN’s electronic health record. A similar model will be considered as in the primary analysis.

##### Realist evaluation

**Fig. 4:**
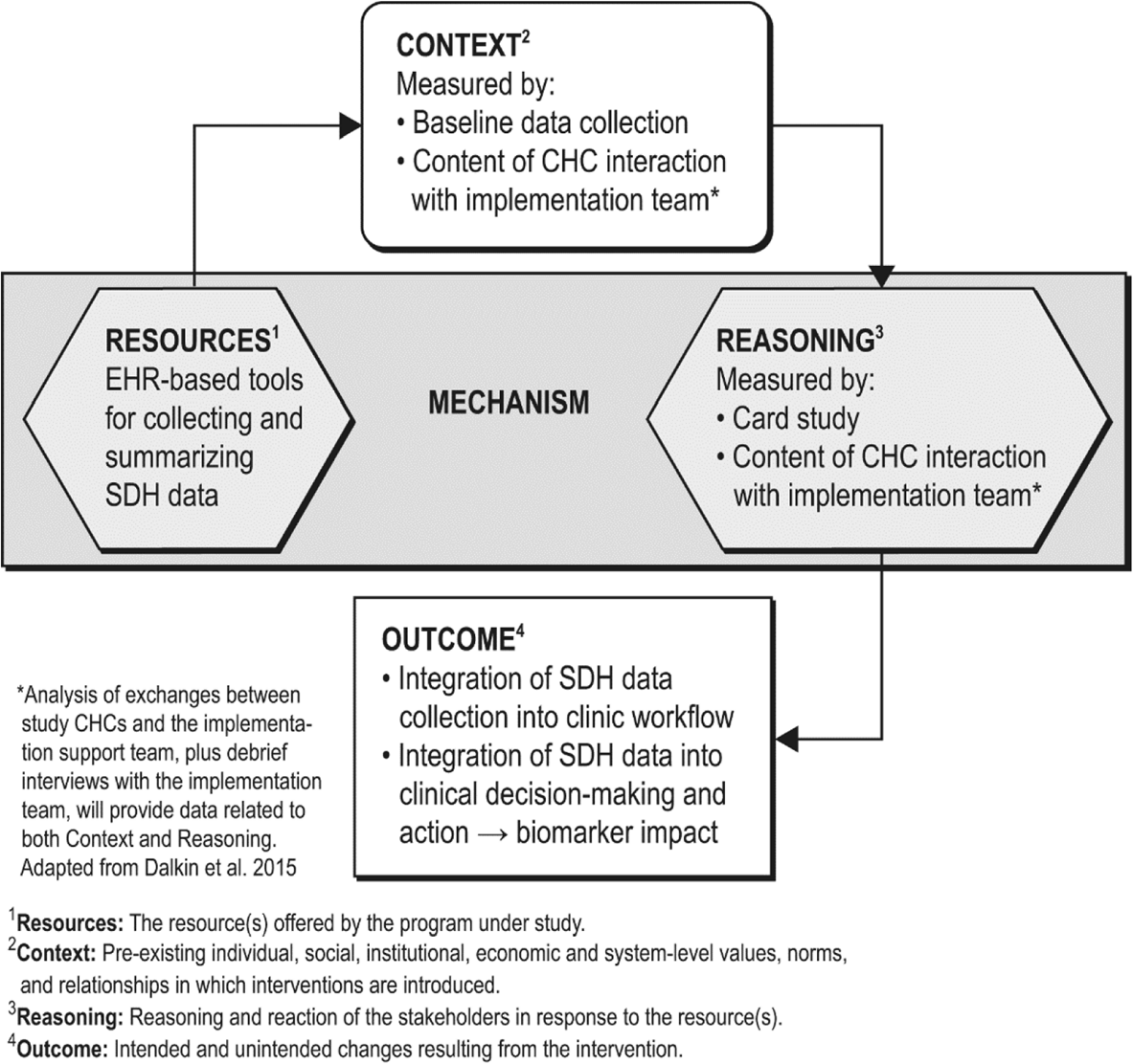
Realist evaluation model—factors influencing intervention impact on outcomes [135, 136, 162]

A key priority for implementation science is identifying the mechanisms by which implementation strategies exert their effects [101, 102]. Realist evaluation clarifies which components of a multifaceted intervention work, for whom, and under what conditions [133], to produce change. Assuming that interactions between contextual and mechanistic factors are key to effective cross-setting translation of interventions [134], realist evaluation conceptualizes intervention outcomes as resulting from a relationship between *context* and *mechanism*: *context* + *mechanism* = *outcome*. We will disaggregate “mechanism” into *resources* and *reasoning*; thus, *mechanism* (*resources*) + *context*➔*mechanism* (*reasoning*) = *outcome* [135]. The goal is to identify context-mechanism-outcome configurations that explain the pathways through which the intervention (tailored implementation support) impacts the systematic collection of social determinants of health data, and the integration of such data into care. This framework will guide data collection and analyses, as below and in Fig. 4.

In this evaluation, the *intervention* is the tailored implementation support (not shown in Fig. 4) and the mechanism (resources) are the electronic health record-based data tools. *Context* can include characteristics of individuals (e.g., roles, attitudes, knowledge), teams (e.g., relationships, team functioning), organizations (e.g., staffing, culture, leadership, resources), and environment (e.g., payor policies, political structures) [134, 136]. Here, context will be measured through (i) a brief baseline survey and (ii) analysis of the exchanges between the clinics and the implementation support team. People (in this case, clinic staff) respond to available resources (mechanism: resources) in different ways [134]. This “response to resources,” or *mechanism (reasoning)*, will be assessed through (i) analysis of interactions between study clinics and the implementation support team (as above) and (ii) a condition-specific card study.

### Data collection

To limit burden on clinics, and mitigate potential Hawthorne effects [137], our realist evaluation will primarily use data collected by the implementation support team in the course of its regular activities. The card study (see below) is the one exception.

### Baseline survey

Shortly before each wedge of community health centers starts the intervention, the operational champion at each clinic will complete a baseline survey. Lacking validated, easily implemented methods for assessing clinics’ readiness to adopt practice changes, we developed a brief baseline survey specifically designed to assess some aspects of readiness as related to adoption of social determinants of health documentation/action. Informed by the building blocks of primary care, it assesses the clinics’ status in empanelment and team-based care, as well as their access to community health workers/social workers/behaviorists; external policies/incentives that might impact results; recent major disruptive events; other clinic initiatives; and payment models. The survey is available in Additional file 2.

### Content of community health center interaction with implementation team

As noted earlier, during each study wedge clinical and/or operational champions from each clinic (as well as any other staff member that is interested in attending) will participate in monthly organization-specific coaching calls and bi-monthly webinars/peer support conversations. With permission, these discussions will be recorded and transcribed. We will track which community health center staff attended these discussions. At monthly calls with each study clinic, we will evaluate each clinic’s progress per the clinic action plan and ask which intervention components were used that month and by whom. We will also document additional support that the study clinics request. We will collect relevant email exchanges between the implementation team and the study clinics, as well as “trouble-tickets” about the social determinants of health tools, as submitted to OCHIN’s member support system. When each wedge ends, we will record a debrief session with the implementation support team to capture their understanding of implementation at each community health center in that wedge.

As shown in Fig. 4, data from the implementation team interactions with study clinics, primarily in the form of monthly check-ins and office hours, are a key data source for measurement of both *context* and *mechanism: reasoning*. The monthly check-ins are a particularly important data source for the evaluation, as the majority of rich back and forth between the implementation team and clinic staff (questions, conversations, talking through challenges the clinics are facing them and brainstorming ways to address those challenges) happens during these organization-specific interactions.

### Card study

We will measure the impact of social determinants of health data on point-of-care decision-making via an electronic health record-embedded card study focused on clinical action (care decisions/referrals) at encounters with patients in each clinic’s target population. Two providers at each study clinic, identified based on how often they see patients that the clinic is targeting for social determinants of health screening, will be recruited by the clinic’s operational champion to complete a < 1 min survey on all patients in the target population seen in a 3-week period. The provider will complete a “card” after the encounter with a targeted patient, which will ask (1) whether/how social determinants of health data informed clinical decisions/actions; (2) how the social determinants of health data was obtained, e.g., via the electronic health record tools?; (3) whether any desired social determinants of health data were unavailable; and (4) estimated time spent looking up social determinants of health data. The questions will not ask for any patient data, and the survey answers will not be saved in the patient chart. We will associate each card with the following information: provider type (MD, SO, PA, NFP, behavioral health), whether the patient was seen by their assigned primary care provider, encounter chief complaint, and whether a completed social determinants of health screen was in the patient’s chart at the time of encounter. These data will be collected ≈ 5 months into the 6-month intervention period.

### Realist evaluation analysis

We will conduct a mixed-methods convergent comparative “case analysis” [138] in which qualitative and quantitative data will be collected concurrently and used to build understanding of the change process in each case (clinic). Data from each case will be “merged” for analysis, then compared *within* and *across* clinics to confirm, expand on, or challenge each site’s findings [116, 138]. Data collection and analysis will be parallel and iterative; analysis will begin at the end of the first wedge and continue as data from each wedge are collected. A grounded theory approach [139–141] and immersion-crystallization process [142] will be used to engage deeply with the data and identify emergent themes [143] that will be categorized into context, mechanism, or outcome. Potential configurations of data in these categories will be proposed, then refined as data collection continues, to identify context-specific intervention components that enable effective implementation of social determinants screening documentation/action [144, 145].

## Discussion

Myriad national initiatives are underway to begin clinic-based social determinants of health documentation/action. These efforts will likely encounter barriers similar to those associated with adoption of any practice change involving new workflows/electronic health record functionalities, plus barriers specific to social determinants of health activities [77]. However, little empirical evidence guides this implementation; to our knowledge, no previous studies have examined the implementation strategies needed to support adoption of social determinants of health-related practice changes in any setting [91]. Even the Centers for Medicare and Medicaid Services’ innovative Accountable Healthcare Communities initiative [146], designed to test “… whether systematically identifying and addressing the health-related social needs … will impact health care costs and reduce health care utilization,” does not focus on the support needs associated with implementing these activities. The study described here will identify strategies for helping community health centers adopt electronic health record-based documentation of patient-reported social determinants of health needs and actions to address those needs [28, 47, 63, 64]. We will document how this support can be tailored to meet local needs and the resources and competencies needed to do so. We chose to test support from a centralized, remote team for its scalability.

Using rigorous methods, the study will also yield important knowledge to dissemination and implementation science as follows:

- We will test the effectiveness of a set of evidence-based implementation strategies which have helped community health centers adopt new workflows/tools in prior research [4, 20, 98, 115–126, 147–157], but that have not been assessed in the context of electronic health record-based social determinants of health documentation/action, either in isolation or in combination. We are not aware of other formal studies of implementation strategies needed to support adoption of this important practice change, despite the need for such guidance.
- We will assess how interdisciplinary implementation teams support practice change [158]. We will track the competencies that the team uses to help the study clinics (e.g., knowledge of electronic health record systems), plus any competencies that the team identifies as needed, and how those needs were met. We recognize that with our tailored strategy, some clinics will need and receive more intensive support. We will document this carefully on our process evaluation by tracking what strategies are needed and provided, and how much time the implementation team spends on each clinic, and overall, to provide the support that is needed.
- This study will yield information on how to tailor implementation support strategies to meet local needs [96, 98, 100, 116]. The step-by-step clinic action plan is designed to be a focused, pragmatic tool that both guides study clinics’ change implementation and enables tracking the specific implementation supports provided at each step, and how this support is tailored. Such detail about tailoring of implementation strategies is rarely reported [117, 159, 160]. Rather than estimating implementation barriers a priori, this approach focuses on the implementation strategy changes that are needed in practice. By documenting where a given clinic gets stuck within an overall shared approach, and the subsequent impact of additional support provided, our findings could have relevance both for specific social determinants of health-related implementation approaches and for other implementation efforts involving tailored support.
- The realist evaluation approach is increasingly used to evaluate complex interventions [135, 136, 161] and is well-suited to pragmatic implementation research due to its emphasis on the impact of context. The focus on identifying the context-specific causal mechanisms through which the tailored support impacts clinic uptake will facilitate appropriate adaptation of successful support strategies to other settings. Furthermore, identifying such causal mechanisms is an implementation science priority, as such mechanisms are infrequently reported.

## Conclusion

Despite the known health impacts of social determinants of health, and a national movement urging healthcare providers to identify and act on patients’ social determinant-related needs, little is known about how to help community health centers adopt social determinants of health documentation/action. By learning whether and how scalable, tailored implementation strategies help community health centers adopt these changes, the proposed study will yield timely guidance to community clinics nationwide.

## Additional files

Additional file 1: Guide to social determinants of health screening and referral-making using the electronic health record. (PDF 4124 kb)

Additional file 2: Baseline survey. (DOCX 23 kb)

## Abbreviations

OCHIN: [Not an acronym]
PRAPARE: Protocol for Responding to and Assessing Patient Assets, Risks, and Experiences
RE-AIM: Reach, Effectiveness, Adoption, Implementation, Maintenance
