# Supplementary material for: Study protocol: a pragmatic, stepped-wedge trial of tailored support for implementing social determinants of health documentation/action in community health centers, with realist evaluation
Source: Implement Sci. 2019 Jan 28;14:9. doi: 10.1186/s13012-019-0855-9 (PMC6348649; doi:10.1186/s13012-019-0855-9)

GUIDE TO SOCIAL DETERMINANTS OF HEALTH SCREENING AND REFERRAL-MAKING USING THE EHR

THE ASCEND PROJECT

KAISER PERMANENTE®
Center for Health Research

OCHIN

A Guide to Social Determinants of Health Screening and Referral-making Using the EHR

This is a guide to implementing social determinants of health (SDH) screening, and taking action on SDH-related needs, using tools in your EHR.

Your clinic's 'SDH Champion' should use this guide as a roadmap.

This guide is organized by the five steps involved in implementing SDH screening and referral-making, with tools to help with each step:

- **Step 1: Create an SDH Team** (Tools: Leadership orientation materials)
- **Step 2**: **Identify clinic goals** (Tools: Recommendations, key considerations, decision tools)
- **Step 3: Create an SDH Plan** (Tools: Tips, considerations, workflow planning tools, rollout planning tools, how to use the EHR tools for SDH)
- **Step 4: Train clinic staff** (Tools: Staff orientation guide, orientation slide deck, email template)
- **Step 5: Roll out, then revise the SDH Plan** (Tools: PDSA cycle worksheet, checklist, tips)

We will provide your SDH Champion with materials at each step, as needed.

The OCHIN Implementation Support Team can help you use this guide. Please contact Julianne Bava at 503-943-5774 or ASCEND@ochin.org**. The Implementation Support Team office hours will provide additional help from the OCHIN team and from other CHCs that are implementing SDH screening and referrals.**

Implementation Steps

Kudos to your clinic for wanting to start screening for social determinants of health (SDH), or wanting to improve your current efforts to do so. SDH can seriously impact patients' ability to act on care recommendations, and to reach their full health potential. You are taking on an important challenge!

As part of the ASCEND study, you will receive assistance (from an implementation support team and your peers) at each step of starting / scaling up your SDH screening efforts. The SDH screening adoption steps are listed below. You can use this list as a roadmap, or adapt it to meet your clinic's needs.

Clinic: ______________________________

Date: ______________________________

Name of person entering data: ______________________________

SDH screening adoption step	Tasks needed for this step	Date completed / support needed
**Step 1. Create a 'SDH Team.'**	Obtain leadership support for SDH screening.	
	Identify a clinician champion (CC) for SDH screening adoption.	
	Identify a project champion (PC); this may be the CC if desired.	
	Give the champion(s) dedicated time for SDH efforts, including contact with study team.	
**Step 2. Identify clinic goals**	Identify your clinic's goals for SDH screening, and which patients you want to screen	
**Step 3. Create a 'SDH Plan.**	Create a workflow plan for SDH data collection and review, and (if desired) SDH action.	
	Create a rollout plan and a plan for tracking your clinic's SDH screening adoption.	
**Step 4. Train clinic staff in the 'SDH Plan.'**	Orient clinic staff (e.g., at a staff meeting, via email, etc.).	
	If changes are made to the plan, orient staff to the changes.	
	Train new staff as needed.	
**Step 5. Roll out, then iteratively revise the 'SDH Plan'**	Start rollout.	
	Review your clinic's SDH screening rates on a regular basis. Use this information to improve adoption of your SDH Plan.	

Implementation Task Timeline

Project month	Led by	Task
1-6	Clinician Champion (CC)	Encourage SDH Plan adoption, answer questions as needed
		Help SDH project champion as needed
	SDH Project Champion (PC)	Monthly calls with OCHIN implementation team
		Attend bi-weekly Office Hours
		Track clinic's progress (online survey)
1-2	Clinic Leadership	Identify clinic's CC and / or PC (Step 1)
	PC & CC	Orientation webinar / first office hours for champions (Step 1)
	Clinic Leadership /PC & CC	Identify clinic's SDH screening goals (Step 2)
	PC & CC	Develop clinic's SDH Plan (Step 3)
	PC (with input from CC)	Develop SDH screening and data collection workflows (Step 3)
3-4	All clinic staff	Orientation to clinic's SDH Plan / SDH EHR Tool Training (Step 4)
	PC	Orient new staff to SDH Plan (Step 4)
	PC	Roll out the 'SDH Plan' – start SDH screening (Step 5)
5-6	PC	Revise the SDH plan as needed (Step 5)

Reference/Resource List

References on SDH:

1) National Academies of Sciences, Engineering, and Medicine. *Accounting for Social Risk Factors in Medicare Payment: Identifying Social Risk Factors*. Washington, DC: The National Academies Press; 2016. Available at: https://www.nap.edu/catalog/21858/accounting-for-social-risk-factors-in-medicare-payment-identifying-social.

2) World Health Organization. 2017. *Social determinants of health: About social determinants of health*. Available at: http://www.who.int/social_determinants/sdh_definition/en/.

3) Gottlieb LM, Wing H, Adler NE. A Systematic Review of Interventions on Patients' Social and Economic Needs. *Am J Prev Med.* 2017;53(5):719-729. [PubMed PMID: 28688725] Available at: https://www.clinicalkey.com/#!/content/playContent/1-s2.0-S0749379717302684?returnurl=https:%2F%2Flinkinghub.elsevier.com%2Fretrieve%2Fpii%2FS0749379717302684%3Fshowall%3Dtrue&referrer=https:%2F%2Fwww.ncbi.nlm.nih.gov%2Fpubmed%2F28688725

References on SDH tools for screening and implementation:

4) National Association of Community Health Centers. PRAPARE. Available at: http://www.nachc.org/research-and-data/prapare/. Accessed on June 26, 2018.

5) National Association of Community Health Centers. PRAPARE Implementation and Action Toolkit. Available at: http://www.nachc.org/research-and-data/prapare/toolkit/. Accessed on July 24th, 2018.

6) AAPCHO. Enabling Services Data Collection Implementation Packet. 2018. Available at: http://www.aapcho.org/resources_db/enabling-services-data-collection-implementation-packet/. Accessed on June 26, 2018.

7) Health Leads. Health Leads Screening Toolkit. 2018. Available at: https://healthleadsusa.org/tools-item/health-leads-screening-toolkit/. Accessed on June 26, 2018.

8) Billioux A, Verlander K, Anthony S, Alley D. *Standardized Screening for Health-Related Social Needs in Clinical Settings. The Accountable Health Communities Screening Tool (Discussion Paper).* National Academy of Medicine Perspectives; May 30, 2017. Available at: https://nam.edu/wp-content/uploads/2017/05/Standardized-Screening-for-Health-Related-Social-Needs-in-Clinical-Settings.pdf.

9) Institute of Medicine. 2014. *Capturing Social and Behavioral Domains and Measures in Electronic Health Records: Phase 2*. Washington, DC: The National Academies Press; 2014. Available at: https://www.nap.edu/catalog/18951/capturing-social-and-behavioral-domains-and-measures-in-electronic-health-records.

Reference/Resource List

References on locating community resources:

10) United way Worldwide. 2-1-1. Available at: http://www.211.org/. Accessed on June 26, 2018.

11) National Center for Medical-Legal Partnership (NCMLP). The Medical-Legal Partnership Toolkit. Available at: http://medical-legalpartnership.org/wp-content/uploads/2017/11/MLP-Toolkit-Phases-I-and-II.pdf. Last Updated March 2015. Accessed on June 26, 2018.

Oregon Primary Care Association (OPCA) references:

12) Oregon Primary Care Association (OPCA). Social Determinants of Health (SDH): Tools & Resources. Available at: https://orpca.com/initiatives/social-determinants-of-health/251-sdoh-tools-resources. Accessed on June 26, 2018.

13) Oregon Primary Care Association (OPCA). APCM Learning Exchange. Available at: https://www.orpca.org/initiatives/alternative-care-model/apcm-learning-exchange. Accessed on June 26, 2018.

14) Oregon Primary Care Association (OPCA). Social Determinants of Health (SDH) Screening Sample Workflow: Steps for Non-Clinical Staff Before the Clinical Visit. Available at: https://www.orpca.org/files/OPCA%20SDH%20non-clinical-staff-before-visit.pdf. Accessed on June 26, 2018.

15) Oregon Primary Care Association (OPCA). Social Determinants of Health (SDH) Screening Sample Workflow: Steps for Clinical Staff During the Clinical Visit. Available at: https://www.orpca.org/files/OPCA%20SDH%20non-clinical-staff-during-clinical-visit.pdf. Accessed on June 26, 2018.

16) Oregon Primary Care Association (OPCA). Social Determinants of Health (SDH) Screening Sample Workflow: Steps for Clinical Staff After the Clinical Visit. Available at: https://www.orpca.org/files/OPCA%20SDH%20non-clinic-staff-after-visit.pdf. Accessed on June 26, 2018.

17) Oregon Primary Care Association (OPCA). Social Determinants of Health (SDH) Screening Sample Workflow: Steps for Using a "No Wrong Door" Approach. Available at: https://www.orpca.org/files/OPCA%20SDH%20no-wrong-door.pdf. Accessed on June 26, 2018.

18) Oregon Primary Care Association (OPCA). Social Determinants of Health (SDH) ICD-10 Z Codes. Available at: https://www.orpca.org/files/OPCA%20SDH%20ICD%2010%20Z%20codes%204.27.18.pdf. Accessed on June 26, 2018.

19) Oregon Primary Care Association (OPCA). Food Insecurity Learning Collaborative Summary Report. July 2017. Available at: https://www.orpca.org/Special%20Pops/OPCA%20Food%20Insecurity%20Collaborative%20Learnings_July%202017.pdf. Accessed on June 26, 2018.

20) Oregon Primary Care Association (OPCA). SEA MAR Adult Social History. Available at: https://www.orpca.org/Special%20Pops/SEA_MAR_AdultSocialHistory.pdf. Accessed on June 26, 2018.

21) Oregon Primary Care Association (OPCA). VA Homelessness Screening Clinical Reminder. Available at: https://www.orpca.org/Special%20Pops/VA_Homelessness_Screening_Clinical_Reminder.pdf. Accessed on June 26, 2018.

Other references cited in this document:

22) Aarons G, Ehrhart MG, Farahnak LR. The implementation leadership scale (ILS): development of a brief measure of unit level implementation leadership. *Implement Sci*. 2014 Apr 14;9(1):45. [PubMed PMID: 24731295] Available at: https://www.ncbi.nlm.nih.gov/pmc/articles/PMC4022333/.

23) Aarons G, Ehrhart MG, Farahnak LR, Hurlburt MS. Leadership and organizational change for implementation (LOCI): a randomized mixed method pilot study of a leadership and organization development intervention for evidence-based practice implementation. *Implement Sci.* 2015 Jan 16;10:11. [PubMed PMID: 25592163] Available at: https://www.ncbi.nlm.nih.gov/pmc/articles/PMC4310135/.

24) Aarons GA, Ehrhart MG, Moullin JC, Torres EM, Green AE. Testing the Leadership and Organizational Change for Implementation (LOCI) Intervention in Substance Abuse Treatment: A Cluster Randomized Trial Study Protocol. *Implement Sci.* 2017 Mar 3;12(1):29. [PubMed PMID: 28253900] Available at: https://www.ncbi.nlm.nih.gov/pmc/articles/PMC5335741/.

25) American Academy of Family Physicians (AAFP). The EveryONE Project Screening Tools and Resources to Advance Health Equity. Available at: www.aafp.org/patient-care/social-determinants-of-health/everyone-project/tools.html. Accessed on June 26, 2018.

26) Centers for Medicare & Medicaid Services. Accountable Health Communities Model. Available at: https://innovation.cms.gov/initiatives/ahcm. Last updated May 3, 2018. Accessed on June 7, 2018.

27) McGinnis JM, Williams-Russo P, Knickman JR. The case for more active policy attention to health promotion. *Health Aff (Millwood).* 2002;21(2):78-93. [PubMed PMID: 11900188] Available at: https://www.healthaffairs.org/doi/abs/10.1377/hlthaff.21.2.78?url_ver=Z39.88-2003&rfr_id=ori%3Arid%3Acrossref.org&rfr_dat=cr_pub%3Dpubmed.

28) Pruitt Z, Emechebe N, Quast T, Taylor P, Bryant K. Expenditure Reductions Associated with a Social Service Referral Program. *Popul Health Manag.* 2018 Apr 17. [Epub 2018 Apr 17] [PubMed PMID: 29664702] Available at: https://www.liebertpub.com/doi/abs/10.1089/pop.2017.0199.

29) Center for Health Care Strategies, Inc. (CHCS). Screening for Social Determinants of Health in Populations with Complex Needs: Implementation Considerations. October, 2017. Available at: https://www.chcs.org/media/SDOH-Complex-Care-Screening-Brief-102617.pdf. Accessed on June 7, 2018.

30) Sabo S, Ingram M, Reinschmidt KM, Schachter K, Jacobs L, Guernsey de Zapien J, Robinson L, Carvajal S. Predictors and a framework for fostering community advocacy as a community health worker core function to eliminate health disparities. *Am J Public Health.* 2013 Jul;103(7):e67-73. [PubMed PMID: 23678904] Available at: https://www.ncbi.nlm.nih.gov/pmc/articles/PMC3682609/.

Step 1: Create an ‘SDH Team’

The first thing you need to do is create your SDH Team.
This is a guide for clinic leaders and SDH champions.

Clinic Leadership Orientation 2

Draft Email from Leadership to Staff 4

Clinician Champion / SDH Project Champion Orientation 5

Step 1: Clinic Leadership Orientation

What are social determinants of health (SDH)?

- SDH are the conditions in which people live and work. They profoundly impact health risks and outcomes, and the ability to act on care recommendations. It is estimated that ***SDH account for about 80% of health outcomes*** (40% socioeconomic factors, 30% health behaviors, 10% physical environment); only 20% of health outcomes are attributed to clinical care (McGinnis, 2002) .

- SDH that impact health include: **housing stability, food security, transportation access, childcare access, ability to pay for utilities, stress, social isolation, etc.**

Why should we collect information on and act on our patients' SDH?

- SDH information gives care teams a **more complete picture** of the factors impacting their patients' health, helping them to:
    - Identify and make needed community **referrals** for a given patient
    - Inform and **adjust care plans** as needed
    - Conduct targeted SDH-related **outreach;** provide **focused support** / assistance
    - Conduct better-informed patient-provider **conversations** about barriers to health
    - Boost staff **morale** by encouraging high-quality interactions with patients
    - SDH screening often captures **previously unknown** information

- Panel-level SDH data can also be used to:
    - **Demonstrate CHCs' value** in serving vulnerable populations
    - **Direct resources** toward specific patients or areas of clinic focus
    - Meet or **improve reimbursement requirements** for value-based care initiatives and metrics for Medicaid managed care quality performance efforts

- Addressing SDH **may reduce costs**: In a 2018 study, managed care patients whose social needs were addressed (through a clinic-led referral program) had annual care costs that were $2443 (10%) less than those whose needs were not met (Pruitt, 2018).

> "…the SDH questionnaire opens up tremendous dialogue on several levels and I absolutely love it."
> *– CHC provider*

Have other OCHIN member clinics started collecting SDH data?

- Yes. OCHIN Epic includes the PRAPARE and Accountable Healthcare Communities (AHC) tools located in the Rooming Activity in the Screenings tab, or in the Flowsheets Activity. You can also select specific SDH domains. (There is not a national standard for SDH screening, but PRAPARE and the AHC screening tools are commonly used.) See References.

- As of June 2018, about 39,000 OCHIN patients ave been screened for SDH in 65 clinics.

Step 1: Clinic Leadership Orientation

How can leadership support SDH adoption?

Your role is **critical** to encourage SDH adoption, and can make all the difference in your clinic's success. To leverage your leadership role to support adoption of SDH screening at your clinic:

- **Tell your staff about your plans to enact SDH screening as early (and often!) as possible.** See draft email text, next page.
- **Enthusiastically support** staff in SDH adoption. Positive leadership leads to positive provider attitudes toward adopting new practices.
- Explain the importance of **every staff member's** contribution to your SDH efforts.
- Make sure that appropriate staff have the **time needed** to gather, review, and act on SDH data.
- Create a sense of **excitement and buy-in** around including SDH in care. This may include messaging to clinic staff, the Board, and key partners. Messages might include:
    - What SDH are in general; which SDH *your* clinic will screen for, and why
    - Why structured SDH screening is important to patient health and outcomes
    - What your clinic will do with SDH data (connect to resources, inform clinical decision-making, work for policy changes)
- **Appreciate individuals' contributions**. Acknowledge staff willingness to collect and review patients' SDH information. Consider sending a monthly email to thank staff for the work they are doing to document and act on SDH.
- Try to stimulate **creative thinking and problem-solving**, and encourage different perspectives. For example, when you encounter barriers to SDH screening, use a few minutes at staff meetings to ask your staff to problem-solve these barriers.
- **Inspire and motivate** your staff. Remind them often how important SDH are to your patients' health. Be clear about your clinic's SDH collection goals, and how they relate to your clinic's mission, in repeated communication. *For example:* Remind staff that SDH screening is a priority at every staff meeting for several months, and reinforce this message in regular emails.
- **Share preliminary data** where possible. Displaying the data on how many screens have been conducted shows staff what they are contributing to, and is impactful.
- Be **proactive** in problem solving, **knowledgeable** about the EHR tools that staff can use in SDH work, **supportive** of staff needs – and **persevere** during the implementation process!

One last tip: Consider building partnerships with local social service agencies. This will make it easier to know which agencies welcome SDH-related referrals from your clinic. It can also help you understand what happens to patients after your staff refer them to community services.

* For more on messaging to stakeholders, see Chapter 2: Engaging Key Stakeholders in the PRAPARE toolkit: http://www.nachc.org/wp-content/uploads/2018/05/Chapter-2-5-12-18.pdf.
* For more on building partnerships, please visit Chapter 8: Build Capacity to Respond to Social Determinants of Health in the PRAPARE Toolkit: http://www.nachc.org/research-and-data/prapare/toolkit/

Step 1: Draft Email from Leadership to Staff

If desired, use this email template to inform your clinic staff about your SDH Plan.

Fill out the bold sections with your clinic's information, and customize as desired.

Dear **(Clinic Name)** Staff –

(Clinic Name) is excited to announce that we are going to start systematically collecting information on our patients' social determinants of health (SDH), and taking action to address identified SDH needs.

SDH are the non-clinical factors that profoundly impact health risks and outcomes, and ability to act on care recommendations, such as housing and food insecurity. Collecting SDH information will help give our care teams a more complete picture of the factors impacting our patients' health, and their ability to act on care recommendations.

(Clinician Champion Name) and **(SDH Project Champion)** will lead these efforts and will be available to answer any questions you may have related to SDH activities.

The expected start date for SDH data collection will be **(Date)**. There will be a staff orientation on **(Date/Time)** – please plan to attend.

[Insert text on SDH Plan (e.g. clinic goals, who you plan to screen, which SDH to screen for, how often etc.)]

Our clinic's planned workflow and rollout plan / timeline **(overview)**

If you have any questions and or concerns, please reach out to **(Clinic Champion Name).**

Sincerely,

(Leadership Name with signature)

Step 1: Clinician Champion / SDH Champion Roles and Responsibilities

Thank you for being your clinic's **Clinician Champion / SDH Project Champions!** You will lead your clinic's efforts to start and/or improve existing social determinants of health (SDH) data collection processes, with help from the Implementation Support Team at OCHIN.

- **SDH Project Champion:** Point of contact for the OCHIN study team. The SDH Project Champion will work directly with clinic staff to implement SDH data collection / action, and will oversee all SDH implementation activities at their clinic.

- **Clinician Champion**: Support SDH Project Champion with all SDH activities, actively encourage SDH plan adoption among fellow providers, and answer questions as needed.

If desired, one person can act as both the SDH Project Champion and Clinician Champion.

It is **up to you** to encourage your clinic to implement SDH data collection – to be the SDH 'cheerleaders.' Your role is **critical**. **To help your clinic start (or scale up) your efforts to document patients' social determinants of health, you will receive the following resources:**

To help with ...	You will receive ...	Offered for:	Time commitment
Implementing SDH collection / referral at your clinic	• Office Hours to help with all SDH implementation aspects<br>• Peer support from other CHCs	2x/month for 6 months	1 hour (highly recommend-ed): PC/CC
	• Staff training webinar on implementing SDH screening / review / action using EHR tools	1x: month 2 or 3	1 hour: All staff
Addressing barriers to SDH collection faced by your clinic	• One-on-one calls (or in-person visits if feasible) with OCHIN implementation support team	1x/month for 6 months	1 hour each: PC
Developing your clinic's SDH data collection / referral workflows	• Examples of SDH data collection / review / action workflows<br>• Workflow planning tool<br>• Guides & training on using EHR's SDH Tools<br>• Support from OCHIN implementation support team (see Office Hours)	1x when need-ed, provided by OCHIN implementation support team	Estimated time to develop your clinic's workflow: 2-4 hours, once: PC/CC
Testing & revising your clinic's SDH workflow / workflow adoption	• Rollout planning tool<br>• Guide to testing and revising workflows<br>• Monthly reports on SDH data collection rates<br>• Support from OCHIN implementation support team (see Office Hours)	Up to 6 months, as needed	Estimated time to test & revise your workflow: 1-2 hours / month over 6 months, or until workflow is adopted: PC
Orienting new staff to clinic's SDH Plan	• Orientation / training materials that the clinic can use to train new staff	As new staff join clinic	1 hour / new staff member as needed: PC
Tracking your clinic's SDH implementation progress overall	• A tracking tool to help you monitor your implementation progress	Up to 6 months, 2x / month	30 minutes / week (required): PC

GUIDE TO SOCIAL DETERMINANTS OF HEALTH SCREENING AND REFERRAL-MAKING USING THE EHR

THE ASCEND PROJECT

KAISER PERMANENTE®
Center for Health Research

OCHIN

Step 2: Identify Your Clinic's Goals for SDH Documentation

The next step is to decide why your clinic wants to conduct SDH screening, and thus which and how many patients you want to screen for which SDH measures. This is a guide for the SDH Champion on how to make these decisions.

Recommendations	3
Key Considerations	4
Decision Tool	
a. Why do you want to screen your patients for SDH?	5
b. Which patients do you want to screen for SDH?	6
c. How many patients do you want to screen?	7
d. Which SDH do you want to screen for?	7

If you need help with any of the aspects of this step, the OCHIN Implementation Support Team can help you. Please contact Julianne Bava at 503-943-5774 or ASCEND@ochin.org. The Implementation Support Team office hours will provide additional help from the OCHIN team and from other CHCs that are implementing SDH screening and referrals.

Step 2: Recommendations

Your clinic goals for SDH screening will determine which patients you want to screen for SDH, which SDH measures to screen for, how often to screen patients, and how many patients you hope to screen.

There are no wrong choices when setting these goals. Your goals should reflect: 1) How you want to use patients' SDH information, and 2) What is best for your clinic.

The Decision Tool on the next few pages will help you think about:

- Potential uses for SDH data
- Which patients you might want to target for SDH screening
- How many patients you want to screen
- Which SDH measures you might want to screen for, and how often

When developing your clinic's SDH screening goals, it can help to:

- Consider **how you want to use the SDH data** when choosing target patients, SDH measures, and other clinic goals related to SDH. There are no national standards about which patients to screen for which SDH in what timeframe. Therefore, your SDH screening goals will be driven by what makes sense for your clinic, and how you want to use the SDH data. For example, if SDH data will be used to understand areas of social need, screening a sample of patients would be adequate. However, if the data are being used to enable targeted outreach, you will want to screen all targeted patients for SDH.
- Start by screening a small group of patients, then scale up once workflows are working well. And / or start by screening for just a few SDH measures; add others when you are ready. For more on how to roll out your SDH Plan, see *Step 3: SDH Rollout*.
- Choose a target population based on **routine, easy-to-identify visits** (*e.g.*, annual physicals, sliding scale re-authorization visits, new patient visits, visits where other annual screenings are conducted). This will help staff identify which patients to screen, and help you track your success at screening your target patients.
- When deciding which SDH measures to screen for, *remember* that the EHR's Screening Section options let you choose specific SDH, or specific groups of SDH (e.g., PRAPARE or the Accountable Healthcare Communities questions). Again, there is no standard for which SDH questions to ask or how to ask them. The domains in your EHR are from PRAPARE Plus and the AHC questionnaire.

Step 2: Key Considerations

Consideration:	Does this apply to me? How?
**Available community resources**<br><br>*E.g.: Support groups, food banks, housing services, and other community programs. You may want to limit screening to SDH for which there are local resources.*	
**Existing clinic resources / partnerships**<br><br>*E.g.: If your clinic has a partnership with local legal services, or an on-site social worker, it might impact what you screen for.*	
**Known barriers or areas of need in your patient panel or community**<br><br>*Does your community have needs that your clinic wants to highlight, or quantify?*	
**Alignment with other clinic priorities**<br><br>*E.g.: Clinic has a grant to screen patients for relationship violence, or is already targeting poorly controlled diabetes, etc.*	
**Staff resources and time commitment**<br><br>*Consider the potential impact of more or less frequent screening; consider the time it will take to screen for multiple SDH needs.*	
**Screening frequency**<br><br>*Do you want to screen for all SDH at the same time (e.g., annually), or screen for some more often than others? Are you participating in any programs that require screening for certain SDH on a specific schedule*	
**Existing SDH screenings**<br><br>*E.g.: If you already screen for intimate partner violence, can you add other SDH at that workflow step?*	
**Existing clinic strategic priorities**<br><br>*E.g.: Clinic has prioritized improving care for high ED utilizers; would screening for SDH help in this population?*	

Step 2: Decision Tool

Identifying your clinic's goals for SDH documentation will help you decide: 1) which patients to screen for SDH; 2) which SDH to screen for, and how often; and 3) how your clinic intends to use the collected SDH data.

a. **Why do you want to screen your patients?**

Review these potential uses for SDH data; check those that apply to your clinic's goals at this time. *If your goals for SDH screening change, consider whether / how that affects which patients you screen, how often, and for which SDH.*

	**1. To provide contextual information that could impact individual patients' treatment plan**	***Prioritize*** **the uses of SDH data for your clinic, if desired:**
☐	**Inform treatment, care planning; know what is affecting patients**<br>*E.g.: Change homeless patient's rx to one that doesn't require refrigeration*	
☐	**Identify & make needed social service intervention referrals_**<br>*E.g.: Refer patient with diabetes, who lacks healthy food, to food bank*	
	**2. To understand areas of need in our clinic / community**	
☐	**Support *organizational* changes - Identify needed staff, allocate resources_**<br>*E.g.: Ensure that a social worker is available to address patients' experience of relationship violence; use SDH data to decide where to locate a new Community Health Worker staff position*	
☐	**Support *community* changes - Provide data for advocacy**<br>*E.g.: Inform local government about need for housing resources*	
☐	**Creating new partnerships with new / other community agencies**<br>*E.g.: Data on patients' legal needs drives creation of medical-legal partnership*	
	**3. To conduct targeted outreach ("Segmentation" of your patient population)**	
☐	**Enable targeted outreach to vulnerable patients**<br>*E.g.: Identify patients with transportation barriers (e.g., those in communities with little public transportation), and refer them to transportation assistance*	
☐	**Prioritize management of complex patients**<br>*E.g.: Community Health Worker identifies patients with social needs for care management program*	
	**4. Respond to external requirements**	
☐	**Conduct screening as required by our health system, state, ACO, etc.**<br>*E.g.: Our CCO requires screening for housing needs.*	

Step 2: Decision Tool

b. **Which patients do you want to screen for SDH?** Pick all that apply. Leave rows blank if not relevant.

Potential patient groups for target for SDH screening	Screen these as soon as you start SDH screening, or later (when)?
**All of your clinic's patients or just a subset**<br>□ All patients, as time allows (skip to section III)<br>□ A subset of our patients (complete the rest of this section)	
**Patients seen at all visit types, or just some visit types?**<br>□ All visit types □ New patient visits □ Non-urgent visits<br>□ Routine annual visits □ Wellness visits □ Other visit type: __________	
**Patients seen by all providers or just selected providers / teams?**<br>□ All providers / teams<br>□ Just some providers / teams: ________________	
**Patients seen at certain days of the week?**<br>□ All days □ Certain days only: ______________________________	
**Gender**<br>□ Men □ Women □ Other	
**Age**<br>□ 0-5 □ 6-12 □ 13-18 □ 19-50 □ 51-65 □ >65 □ Other:__________	
**Target patients with chronic or comorbid medical conditions?**<br>□ No □ Yes: which conditions?<br>□ DM □ CVD □ Mental / behavioral health □ Other:_______	
**Target patients with substance use disorders?**<br>□ No □ Yes: Which disorders? ______________________	
**Target patients with specific utilization patterns?**<br>□ No □ Yes, defined as: _____________________	
**Pregnant women**<br>□ No □ Yes, this is a specific criteria for screening	
**Participants in other clinic initiatives**<br>□ No □ Yes: which? ____________________	
**Patients being screened for other needs?**<br>□ No □ Yes: which one? (e.g., SBIRT, PHQ): _____________________	
**Other factors or patient characteristics**<br>□ No □ Yes: which? ____________________	

Step 2: Decision Tool

c. **How *many* patients do you want to screen for SDH?**

**In first 6 months:**	□ All	□ _____% of targeted patients	□ ____ # of targeted patients
**In first 12 months:**	□ All	□ _____% of targeted patients	□ ____ # of targeted patients
**Annually:**	□ All	□ _____% of targeted patients	□ ____ # of targeted patients

d. **Which SDH do you want to screen for, and how often?**

NOTE: The SDH options listed here are in the Epic EHR's Screening Section.

Check which SDH you want to screen for / record in the EHR		How often do you want to screen for this? (e.g., annually, 1st visit, etc.)
All SDH Domains (a.k.a., OCHIN Epic SDH/PRAPARE-plus)		
All AHC (CMS Accountable Healthcare Communities)[a]		
Or individual SDH domains?		
**SDH Domains**	In AHC?	
Health Literacy		
Education level[b]	Yes	
Financial resource strain[b]		
Housing situation	Yes	
Food	Yes	
Transportation	Yes	
Utilities	Yes	
Physical activity[b]		
Social isolation[b]		
Stress[b]		
Relationship safety[b]	Yes	
Help Desired		

[a] Required core domains for Oregon clinics taking part in the CMS / AHC project

[b] Recommended by IOM committee for inclusion in EHRs. Other domains recommended by the IOM that are already collected elsewhere in OCHIN Epic include race/ethic group, depression, tobacco use, and alcohol use.

Your next step is to create an SDH Plan. This includes choosing your workflows for SDH data collection and SDH data review and referral-making, and deciding how you want to roll out these workflows.

GUIDE TO SOCIAL DETERMINANTS OF HEALTH SCREENING AND REFERRAL-MAKING USING THE EHR

THE ASCEND PROJECT

KAISER PERMANENTE®
Center for Health Research

OCHIN

Step 3: Create an 'SDH Plan'

Your next step is to create an SDH Plan. This includes choosing your workflows for SDH data collection and SDH data review and referral-making, and deciding how you want to roll out these workflows. This guide will help your SDH Champion make these decisions.

Please note that all workflow tools are divided into those for SDH data collection (aka documentation), and those for SDH data review and referral-making (aka action).

SDH Data *Collection*

Considerations based on other CHCs' experiences 3

Pros and cons to different SDH data collection workflow options 5

Workflow planning tool 7

SDH Data *Review / Referral-making*

Considerations based on other CHCs' experiences 9

Workflow planning tool 11

SDH Rollout

Key considerations 13

Rollout planning tool 14

Using the Social Determinants of Health EHR Tool in Workflows 15

How to Create and Maintain a Community Resource List 18

If you need help with any of the aspects of this step, the OCHIN Implementation Support Team can help you. Please contact Julianne Bava at 503-943-5774 or ASCEND@ochin.org. The Implementation Support Team office hours will provide additional help from the OCHIN team and from other CHCs that are implementing SDH screening and referrals. For step-by-step instructions on using the SDH tools in Epic, see ASCEND SDH user guides in Ella.

Considerations Based on Other CHCs' Experiences

Which patients should we screen? How often?

Q: Which patients should we target for SDH screening?

- There is no right way to do this; your clinic can choose which patients you want to screen. The Step 3 guide will walk you through your options.
- Other clinics have targeted: Patients at annual (Medicare) exams; new patients; one provider's panel; patients 65+; patients in care management programs; homeless patients; patients also referred to behavioral health care or the CHW or social worker.

Q: Should we try to screen patients that are difficult to reach, through outreach?

- Some CHCs found a low return on invested time when SDH screening was conducted via outreach to patients who hadn't been seen in a long time.

Q: How often should we re-screen patients?

- There are no rules for this. Some CHCs find it hard to screen more often than annually.

What are important workflow and staffing considerations for SDH screening?

Q: Which staff roles are most appropriate for the SDH workflow?

- This will depend on your clinic's structure and resources. Be sure that staff assigned to SDH activities have the needed time, workload, expertise, and comfort level.

Q: What should the PCP's role be?

- At a minimum, the SDH workflow should let the PCP review previously collected SDH data (and any follow-up action taken). PCPs often want to hear about new SDH information or actions, because they may use that information to inform care plans.
  - PCPs can also; refer patients to appropriate team members for further help, as needed; deliver personalized messages about how to access community resources; and ensure that resource contact information is added to the AVS.

Q: We would like MAs to administer SDH screening during rooming - will this work?

- Yes, sometimes rooming staff can: review completed SDH screening questionnaire and determine patient needs; crosswalk needs with known resources in your community, and come up with a plan to help manage the patient's SDH needs; counsel patient during the visit; and / or assist with documentation and follow-up.
- However, it can be hard to administer the full SDH screening during rooming; verbally asking the full questionnaire in person can take up to half an hour.

- **TIP:** Add SDH screening to routine visits (annual physicals, drug screenings, paperwork re-authorizations, etc.) rather than targeting patients based on other characteristics. This will reduce perceived stigma, and facilitate identifying patients for screening, re-screening patients on a schedule, and ability to scale-up.
- **TIP:** Be flexible about modifying your target population as your workflow is revised.
- **TIP:** Do outreach as part of your SDH workflow, but not as your primary focus.
- **TIP:** Think about: Who is or might be interested? Who has the right knowledge and skills or is willing to learn? Who has the time?
- **TIP:** If your clinic plans to administer only certain sections of the SDH questionnaire, doing so at rooming may be possible.
- **TIP:** If you want to administer the screening in person, assign it to a staff person who can spend more time with each patient.
- **TIP:** It is also now possible to let patients enter their own SDH data directly into the EHR, in the exam room. See *How to Document EHR Data.*

Q: Are personal interactions between patients and staff needed at every step of the process?

- ***Screening***: Some CHCs administer the SDH questionnaire in person; others ask patients to fill it out and then have staff enter the data into the SDH flowsheet.
- ***Referral***: Some CHCs find that giving patients community resource information based just on SDH questionnaire responses is less effective than in-person follow-up to positive SDH screening results.

- **TIP:** Use the method that best fits your clinic's workflow, resources and culture.

How can we head off potential challenges to adopting SDH screening and referral-making?

Q: What changes can we make to facilitate adoption of SDH screening?

- Often it is non-clinical staff who collect and act on SDH. These staff may have varying degrees of comfort and skill with the EHR, so be sure to:
  - Train them in use of the EHR tools (provide at-the-elbow support, as necessary).
  - In the Rooming Activity, wrench the Screening tab to appear where desired.
  - In the Flowsheet Activity:
    - 'Wrench' the SDH Flowsheet so it always appears.
    - Find Tips and Tricks in the sidebar to find SmartLinks to incorporate SDH in your progress note.

- **TIP:** If you are creating and using Preference Lists for SDH referrals, ensure that staff who will use Preference Lists have security access to them!

Q: What struggles are common to CHCs doing SDH data collection?

- You can use Reporting Workbench to identify patients who are targeted for SDH screening, by noting them as having a 'Questionnaire Pending.' This will tell front desk or rooming staff who to screen. See *Using Reporting Workbench to Identify Targeted Patients*, later in this step. Or you can target patients who are easy to identify routinely – for example, by including the paper SDH questionnaire in pre-set screening packets (e.g., new patients, annual physicals, annual insurance reauthorization).
- It can be hard to find the time needed to do this work, among many other priorities.

- **TIP:** Align SDH screening with other clinic efforts / initiatives and quality improvement metrics, as possible. Who might be interested? Who has the right knowledge and skills or is willing to learn? Who has the time?

Step 3 SDH: Data Collection
Pros and cons to different SDH data collection workflow options

Option	PROS	CONS
Patient answers SDH questions via **patient portal, before** the day of the encounter	• SDH data automatically sent to Epic.<br>• Reporting Workbench can send **batch emails** to ask patients to complete SDH form in the portal. You can time these emails as needed. (Will apply to AHC clinics.)	• Not all patients have a portal account; a complementary method will be needed.<br>• Does not allow screening for single SDH questions – just PRAPARE or AHC.
Patient answers SDH questions via **patient portal, in the waiting room**	• SDH data automatically sent to Epic.<br>• You can sign patients up for the portal on a tablet / computer, or by having them download the portal's smartphone app. (May help with portal adoption rates.) Once the patient has a portal account, email them the SDH screening link.<br>• 'Questionnaire Pending' will tell front desk staff which patient to screen.	• Not all clinics have tablets. Tablets must be managed by staff, and kept clean.<br>• Not all patients have a smartphone; a complementary method will be needed.<br>• Four steps: 1) patient signs up for portal; 2) staff emails SDH screening link to patient; 3) patient enters data; 4) data sent through portal must be filed before becoming part of medical record.<br>• If patient is late, may not be time for this.<br>• 'Questionnaire pending' requires prior step of sending batch orders for questionnaires to targeted patients.
Patient answers SDH questions on paper, **in the waiting room**	• Does not take up encounter time.<br>• Does not require portal account or smartphone.<br>• If desired, provider can review patient's answers from paper form.<br>• 'Questionnaire Pending' will tell front desk staff which patient to screen.	• Data must be entered by staff, and data entry timing will impact how data can be reviewed in the EHR.<br>• Less privacy in the waiting room.<br>• If patient is late, may not be time for this.<br>• Requires a way to tell front desk staff which patients should receive a paper form. E.g. , the DAR.<br>• 'Questionnaire pending' requires prior step of sending batch orders for questionnaires to targeted patients.
Staff asks SDH questions **in the waiting room pre- encounter**	• Does not take up encounter time.<br>• Data entered in time is ready for provider review, if desired.<br>• Trained staff asks SDH questions.	• 'Questionnaire pending' requires prior step of sending batch orders for questionnaires to targeted patients.<br>• Less privacy in the waiting room.<br>• Data must be entered by staff, and data entry timing will impact how data can be reviewed in the EHR.<br>• If patient is late, may not be time for this.<br>• Walk-in patients may be missed.
Staff asks SDH questions **in a separate office**, before the encounter	• 'Questionnaire Pending' will tell front desk staff which patient to screen.<br>• Does not take up encounter time.<br>• Staff can make referrals at same time.<br>• Data entered in time is ready for provider review, if desired.	• 'Questionnaire pending' requires prior step of sending batch orders for questionnaires to targeted patients.<br>• Could be hard to schedule.<br>• Clinics may not have space for this.

Step 3: SDH Data Collection

Option	PROS	CONS
[PENDING OPTION: Patient enters SDH data directly into EHR, **in the exam room**	• Data entered in real time is ready for provider review.<br>• 'Questionnaire Pending' will tell rooming staff which patient to screen.<br>• Patient privacy.<br>• Epic can be 'locked' so that patient can enter data, without closing the encounter; functionality pending.	• If provider enters room before screening is done, it may not be completed at that visit.<br>• 'Questionnaire pending' requires prior step of sending batch orders for questionnaires to targeted patients.
Staff asks SDH questions **in the exam room**	• Data entered in real time is ready for provider review.<br>• Trained staff asks SDH questions.<br>• Patient privacy.<br>• Staff may be able to bill for SDH screening time. (Ask your billing department.)<br>• 'Questionnaire Pending' will tell rooming staff which patient to screen.	• If conducting full PRAPARE or AHC screening, may not have time.<br>• If patient is late, may not be time for this.<br>• If provider enters room before screening is done, it may not be completed at that visit.<br>• Increases patient time in the exam room.<br>• 'Questionnaire pending' requires prior step of sending batch orders for questionnaires to targeted patients.
Staff asks SDH questions **at the clinic after the provider encounter.**	• The person administering the screening could also refer the patient to needed local resources.	• SDH information is not available during the visit.<br>• Increases the patient's time at the clinic.

WORKFLOW PLANNING TOOL

Use this tool to select your SDH data collection workflow.

This is just a guide! You may want to choose options that are not listed here.

Circle your answers to create a record of your choices; pick as many as apply.

*Date:*______________________

Decision	Likely choices
**1. When will your clinic collect SDH data?**	• Before the visit (MyChart)<br>• During the visit – at check-in, with other screenings<br>• During the visit – at rooming<br>• During the visit – after the encounter (warm hand off)<br>• During the visit – other<br>• After the visit – follow-up call<br>• After the visit – other<br>• Other ___________________________
**2. Who will administer / hand out SDH screening questionnaire?**	• Front desk<br>• Behavioral health staff<br>• Community health worker<br>• Enrollment staff / eligibility specialist<br>• Care manager / coordinator<br>• Panel manager<br>• Patient advocate / navigator<br>• Other ___________________________
**3. What data collection method(s) will your clinic use to collect SDH data?**	• MyChart pre-visit (data is sent to EHR)<br>• MyChart sign-up, data entry at visit<br>• Staff query patient, enter data in EHR flowsheet<br>• Staff query patient, record data on paper, enter into EHR later<br>• Patient completes paper form<br>• Patient completes form directly into EHR in exam room<br>• Patient uses clinic tablet (data is sent to EHR)<br>• Other ___________________________

Step 3: SDH Data Collection

<table>
<tr><th>Decision</th><th>Likely choices</th></tr>
<tr><td>4. If using MyChart: Which patients will be asked to complete screening, when, and how?</td><td>Who?
• All targeted patients
• Next month's visits
• Next week's visits
• Other ______________________
When? (Be sure to align with your clinic's screening goals)
• 1x/year
• 2x/year
• 1st of month
• Pick day of week ________________
• Other ______________________
Which staff person will send batch emails asking patients to complete MyChart form?
• Front desk staff
• Care manager
• CHW / CSW
• MA
• Other</td></tr>
<tr><td>5. If collecting data on paper form: Who will enter these data and when?</td><td>Who?
• Rooming staff
• Front desk
• CHW / care manager
• Behavioral health provider
• Other ______________________
When?
• Before provider encounter – at rooming
• Before provider encounter – other time
• After encounter – daily
• After encounter – every 48 hours
• Other ______________________
Will all answers be data-entered?
• All
• Only some: ___________________</td></tr>
<tr><td>6. Will we document the SDH need in the problem list?</td><td>• No
• Yes, with diagnostic codes
• Yes, other</td></tr>
</table>

Step 3: Data Review / Referral-making

Considerations Based on Other CHCs' Experiences

Q: A patient has indicated they need / want help - what next?

- Some CHCs have the **same staff person** administer the screening *and* provide any indicated referrals at the same encounter.

- Some CHCs use a **warm hand-off** to address **all** SDH needs: *e.g.*, a patient with SDH needs is sent to meet with a social worker, CHW, etc., as soon as needs are identified.
  - **Con:** It can be hard to ensure that the right person is available for the hand-off.
- Some use a **warm hand-off** if a patient screens positive for SDH needs that are **urgent**.

- Some CHCs have the staff person who administers SDH screening / enters SDH data send a ***generic* internal referral** (e.g., 'social need') to a CHW, behavioral health person, etc., for assistance either at the current visit, OR in follow-up after the visit.
  - **Pros:** Expedites referrals; allows staff with time and expertise to work with the patient to determine appropriate follow-up.
  - **Con:** Follow-up can take time, can be hard to address at same encounter.
- Some CHCs have this staff person send a ***specific* internal referral**(s) based on identified needs for assistance either at the current visit, OR in follow-up after the visit.
  - **Pro:** Faster identification of needed resources.
  - **Cons:** Labor-intensive for person making referral. Less customized to patient priorities.

Q: Will we be overwhelmed with the number of positive responses?

- It is likely that the majority of CHC patients will report at least one SDH need. However, only a small percentage of them may desire clinic staff help in addressing these needs.

  - **TIP**: Ask patients if they want clinic assistance with any SDH needs; use the SDH flowsheet to record their response.
  - **TIP**: Recognize that you can work with patients on additional needs at a future appointment. This is an ongoing process; not all needs can / will be solved immediately.

Step 3: Data Review / Referral-making

Q: Our patients often have multiple SDH-related needs – how do I prioritize?

- There is no easy answer. In some CHCs, prioritization is based on patient desires, available resources and staff assessment of need.
- Keep in mind that screening for pre-determined SDH measures can feel more proscribed and less patient-centered than asking the patient what is most important to them, and building on that.

Q: How should we communicate SDH screening results back to the PCP and larger care team?

- *Via EHR:* Use SmartLinks (see Tips and Tricks in the Flowsheet sidebar) to send SDH screening results into the chart note, or to an internal referral, **and / or** add SDH needs to the problem list (see EHR Tools document for suggested documentation codes).
- *Paper or in-person:* Give the PCP the completed paper SDH questionnaire to scan prior to seeing the patient, **and / or** consider using a quick in-person huddle to share important SDH information with the whole care team.

Q: Can we use Reporting Workbench reports to help us track this work?

- Yes. See EHR Tools document for details.

- **TIP:** Explain to the patient that you may not have a solution to all of the patient's needs, but you are attempting to better understand their life. (Sets expectation around clinic ability to address needs.)
- **TIP:** Document in chart notes that certain needs were not met.
- **TIP:** See Oregon Primary Care Association: https://www.orpca.org/files/8%20Principles%20for%20Patient-Centered%20Social%20Determinants%20of%20Health%20Screening.pdf

- **TIP:** Use the SDH Summary to review patients' SDH needs, not the flowsheet.
- **TIP:** One patient-centered practice is to ensure ALL team members touching the patient can see answers recorded. Asking questions twice in the same visit, or in a 6-month period, can be frustrating for the patient.
- **TIP:** See Oregon Primary Care Association: https://www.orpca.org/files/8%20Principles%20for%20Patient-Centered%20Social%20Determinants%20of%20Health%20Screening.pdf

ONE LAST TIP: Make sure that staff who are tasked with making referrals have security clearance to access the EHR's referral tools!

WORKFLOW PLANNING TOOL

Use this tool to select your clinic's SDH data review & action workflows.

This is just a guide! You may want to choose options that are not listed here.

Circle your answers to create a record of your choices; pick as many as apply.

*Date:*______________________

Decision	Likely choices
**1. Which staff member(s) will review *individual* patients' reported SDH needs?**	• Clinician<br>• CHW/CSW<br>• MA<br>• Other ________________________
**2. When will this person review these SDH needs?**	• Before the visit<br>• After the visit<br>• During visit – before PCP enters<br>• During visit – PCP reviews<br>• During visit – after PCP leaves<br>• Other ________________________
**3. How will the reviewer respond to identified SDH needs? How often?**	• Refer to community agencies (external)<br>• Refer to CHW / CSW / navigator / other (internal)<br>• Flag for clinician to make needed referrals (internal or external)<br>• Other ________________________<br>***How often will this occur?***<br>• One time only, post-visit<br>• During ongoing navigation<br>• Other ________________________

**4. When will the reviewer respond to identified SDH needs (e.g., by making referrals to community service agencies)?**	• During the visit, on site<br>• After the visit, by phone or another method<br>• During outreach / panel management<br>***If during outreach, how often?***<br>• Weekly / Monthly / Other ____________________
**5. How will your clinic document SDH referrals made by clinic staff, if at all?**	***We will document with .Smartphrases***<br>• Yes / No<br>***We will document with referrals (priority option = no follow-up needed)***<br>• Yes / No<br>***We will document in chart notes***<br>• Yes / No
**6. How often will you follow up on SDH referrals, if at all? Who will do it?**	• Never<br>• Weekly<br>• Monthly<br>***Who will be responsible for doing follow-up?***<br>• CHW/CSW<br>• MA<br>• Clinician<br>• Other ______________________________________<br>***How?***<br>• Phone call – outreach / follow up<br>• Ask in person at next visit<br>• Other ______________________________________
**7. What will you consider a 'resolved' SDH need?**	• No need reported at next annual screening<br>• Follow-up with patient to confirm need met<br>• Follow-up with service agency to confirm need met<br>• Other _____________________________________

Step 3: SDH Rollout
Key Considerations

1. **Small tests of change** can accelerate adoption of an SDH Plan more than making large- scale changes all at once. Start with one provider, one screening, on one day, to test workflows.
2. **Start small, then expand** once you identify and fix 'bugs' in your SDH Plan. Pick one or two populations of focus, to start. Take what you learn from these, adapt your SDH Plan as needed, then scale up to more patient groups. (In other words, use a Plan-Do-Study-Act cycle to learn before scaling up).
3. **Check small samples of screening rate data**, daily or weekly, to decide how you need to adapt your SDH Plan. (You might also want to review the SDH screening results.) Check in with both high and low performers!
4. Make expanding the rollout a **team effort**, rather than having one person be responsible for making it happen. This will improve buy-in!
5. The rollout planning tool (next page) will help you select a SDH rollout plan.
6. **The Step 5 documents will walk you through how to iterate your SDH Plan as needed, using Plan, Do, Study, Act (PDSA) cycles.**

Examples: SDH rollout plans used by other clinics

Example 1 – Red Clinic:

- SDH collection / review was done among *new patients seen by the lead clinician*. Over two weeks, at team huddles, they identified and corrected glitches in the planned workflows for collecting / reviewing SDH needs data, and referring patients to community resources.
- Then SDH screening was expanded to *all* adult patients seen by this clinician.
- Two weeks later, the team presented their workflow to the rest of the clinic, after which the *whole clinic* started collecting SDH data on all patients, using the tested, revised workflow.

Example 2 – Blue Clinic:

- Clinic leadership developed an SDH Plan for data collection / review / action, and presented it at an all-staff meeting, saying that these workflows would start the next day, clinic-wide.
- Over the next month, the SDH champion identified which teams / providers were / were not screening targeted patients, by looking at weekly Reporting Workbench data. She followed up with low adopters, encouraged them to adopt the SDH Plan, and helped them as needed.
- The clinic's SDH champion continued to review rates of SDH documentation / referral monthly and check in on low-adopting teams.

Example 3 – Yellow Clinic:

This clinic used a formal Plan-Do-Study-Act process to test their SDH Plan. They:

1. Listed the tasks needed to implement their SDH Plan.
2. Implemented the plan within one clinic care 'pod.'
3. After a week, the pod reported on what happened when they implemented the SDH Plan.
4. Clinic leadership / SDH champion used Reporting Workbench reports to review statistics on how many targeted patients the test pod screened and referred.
5. Planned how to modify the workflow, made needed modifications, went back to Step 1.

Step 3: SDH Rollout
Rollout Planning Tool

Use this tool to select your clinic's SDH data collection rollout plan.
This is just a guide! You may want to choose options that are not listed here.
Circle your answers to create a record of your choices; pick all that apply.

Decision	Likely choices
**1. Who will start your SDH plan first?**	• Just one provider / team / pod<br>• Whole clinic<br>• Other ________________________
**2. If starting with one team / pod, how soon after they start the SDH plan will you review their adoption rates?**	• 1 week<br>• 2 weeks<br>• 1 month<br>• 2 months<br>• Will wait until workflows and process solidified (revisit monthly)<br>• Not starting with just one team/pod<br>• Other ________________________
**3. How will you evaluate adoption of your SDH plan in this first group? (Select all that apply)**	• Clinic leadership input<br>• Review SDH screening rates<br>• Team input<br>• Review SDH screening workflow PDSA data<br>• Other
**4. How often will your clinic track your clinic's SDH screening adoption success?**	• Weekly<br>• Monthly<br>• Once a year<br>• Twice a year<br>• Other ________________________
**5. What will your next step be (if you start with a subset of the clinic)?**	• Expand target population<br>• Have another provider / team / pod start the SDH plan; iterate<br>• Have entire clinic start the SDH plan<br>• Other ________________________
**6. How will you evaluate adoption of your SDH plan after this step? (Select all that apply)**	• Clinic leadership input<br>• Review SDH screening rates<br>• Team input<br>• Other

* For more on workflows, see Chapter 5: Workflow Implementation in the PRAPARE toolkit: http://www.nachc.org/wp-content/uploads/2018/05/Chapter-5-5-7-18.pdf and SDH: Tools and Resources on the OPCA website: https://www.orpca.org/initiatives/social-determinants-of-health/251-sdoh-tools-resources

* For more on enabling services, see Chapter 10: Track Enabling services in the PRAPARE toolkit: http://www.nachc.org/wp-content/uploads/2016/08/Chapter10-Track_Enabling_Services_Aug2016.pdf

Using the Social Determinants of Health EHR Tools in Workflows

Before the visit

- Use Reporting Workbench to **identify** patients and assign them an Active Questionnaire Series.
- Then **send** a batch MyChart message and attach the appropriate questionnaire, asking these patients to complete SDH Screening. Use the Mychart message template **OCHIN New MyChart Questionnaire.** This will **note** in the EHR that patients have a pending questionnaire.
- … Or, you can then send a batch of **letters** via USPS with an attached questionnaire for the patient to send back. Include a self-addressed stamped envelope for easy return. It also helps to make an SDH letter template or SmartPhrase with the questionnaire attached. Or, use letter template **OCHIN MyChart Activation-New Questionnaire** so patient can activate MyChart and answer the questionnaire in MyChart.

At check-in

If your clinic is handing out the paper form for SDH screening:

- Front desk staff can use the DAR column (or other alert) to **identify** which patients have an unanswered SDH questionnaire, and should be given an SDH screening form.
- After the patient completes the form, data can be **entered by staff** into the SDH Flowsheet.

If your clinic will have a staff person conduct SDH screenings before rooming:

- Enter data directly into the SDH Flowsheet.

If your clinic will use a tablet to ask SDH screening questions:

- Patients with a smartphone: Send a MyChart Patient Message with the SDH questionnaire. Or, have patient sign up for MyChart on their smartphone, then send a MyChart message.
- If you use the Welcome tablet, the patient can complete SDH screening on the tablet, or a staff person can query the patient and enter data into the tablet. Or, use the tablet to sign the patient up for MyChart and let them complete the screening on MyChart.
- OCHIN clinics in the AHC / CMS project: data can be entered into the AHC tablet, but will not be in the EHR unless the screening is completed in MyChart.

TIPS:

- The OCHIN team can help you set up this Reporting Workbench report to create a patient roster.
- Using this roster to identify patients ahead of time is essential if you want to notify front desk / rooming staff which patients to screen, or track your clinic's SDH screening progress.
- Using MyChart to collect SDH data only works if patients have a MyChart account. You'll need another data collection method to augment this one.
- Be sure to have the patient complete the SDH Screening questionnaire that your clinic uses (PRAPARE, AHC).
- Be sure the SDH data are entered into the EHR in time for review at the encounter, if desired. Your SDH Plan should say who will enter these data, and when. Data entry takes 1-2 minutes.
- Patients can download the MyChart app onto a smartphone.
- After patient signs up for MyChart, a Patient Message must be sent to give them the SDH questionnaire link. This can be a two-step process (1: sign up, 2: send link).
- Data entered through MyChart will go in the record once it has been filed.

For more information see User Guides in Ella: *ASCEND SDH, Identifying Patients who are Targeted for SDH Screening; ASCEND SDH, Documenting and Viewing SDH Data; Sending / Reviewing Patient Questionnaires (video); MyChart: Quickly File Patient Questionnaires to Flowsheet Activity; MyChart Improvement Guide; MyChart Adoption Guide; ASCEND SDH, Document and View SDH Data, Sending / Reviewing Patient Questionnaires; MyChart: Quickly File Patient Questionnaires to Flowsheet Activity; MyChart Improvement Guide; MyChart Adoption Guide.*

Using the Social Determinants of Health EHR Tools in Workflows

At rooming

- Use the *DAR* column (or other alert) to **identify** which patients have an SDH questionnaire pending, and should be given an SDH screening form. (In Rooming Activity, **Patient Questionnaires** in the table of contents shows if there are patient-reported data to be filed).
- In Rooming, use the Screenings tab to enter SDH data, or **enter** SDH data directly in the Flowsheets activity.
- Use the Social Determinants of Health Snapshot Summary to **review** patients' SDH data.
- Use Order Entry (internal referral) or a Preference List to **refer** patients with SDH needs (who desire assistance) to a social worker, CHW, etc., or to **give the patient information about community agencies** that can address their needs.
- Use *SmartPhrases* to document SDH-related referrals

  .SDHDECLINED

  .SDHREFERRALDECLINED

  .SDHHANDOUTS
- **PENDING option:** 'Lock' Epic to let the patient enter SDH responses directly into the EHR, from the exam room.

TIPS:

- The OCHIN team can help you set up the roster through Reporting Workbench in order to flag patients as 'Questionnaire pending.'
- Make sure that the person making internal referrals has security clearance to sign the referrals.
- Selecting community agencies in SDH Referral preference lists sends agency information to the After Visit Summary, which can be viewed in MyChart.
- Pending option: Responses will then be reviewed from the receiver's In Basket, and filed to their EHR.

For more information see User Guides in Ella: *ASCEND SDH, Document and View SDH Data; ASCEND SDH, Make and Review Community Referrals*

During the encounter

- Use the *SDH Snapshot Summary* to **review** patients' SDH data, then:
  - Make **referrals** to clinic staff or outside agencies to address the patient's SDH needs, and / or
  - **Adapt** the care plan to accommodate the patient's needs.
- Use Order Entry or your clinic's Preference List to **refer** patients with SDH needs (who desire assistance) to a social worker, CHW, etc.
- Use *SDH Referrals* (if your clinic built them) to **refer** patients with SDH needs to community agencies that can address their needs.
- **Enter** SDH data into the Problem List.
- Use *SmartPhrases* to **document** SDH-related referrals

.SDHDECLINED

.SDHREFERRALDECLINED

.SDHHANDOUTS

TIPS:

- In the *SDH Snapshot Summary* you can hover over a given SDH need to get more information.
- Make sure that the person making internal referrals has security clearance to do so.
- Selecting community agencies in *SDH Referrals* sends agency information to the After Visit Summary, which can be viewed in MyChart.

For more information see User Guides in Ella: *ASCEND SDH, Make and Review Community Referrals; ASCEND SDH, Document and Code SDH Diagnoses*

After the encounter / between encounters

- Use the Social Determinants of Health Snapshot Summary and chart review to **review** a patient's SDH needs and whether/when they received referrals to address those needs.
- Use your site's Preference List in Order Entry to **refer** patients with SDH needs (who desire assistance) to a social worker, CHW, etc.
- Use the SDH Referrals (if your clinic built them) to refer patients with SDH needs to community agencies that can address their needs.
- Use Reporting Workbench to **follow up** with patients who had a positive SDH screening and wanted clinic assistance with their SDH need.
- Use Reporting Workbench to identify patients who are targeted for SDH screening and do not have a pending visit, for **outreach**.

TIPS:

- Make sure that the person making referrals has security to sign the referrals.
- Selecting community agencies in SDH Referral order sends agency information to the After Visit Summary, which can be viewed in MyChart.

For more information see User Guides in Ella: *ASCEND SDH, Make and Review Community Referrals*

How to Create and Maintain a Community Resource List

There are several options for how your community resource list might be created.

Option	Pros	Cons	Tips / considerations
1. Create and maintain a clinic binder or spreadsheet with information on local social service organizations. List local agencies to which your teams often refer patients.	Many CHCs already have a community resource binder, spreadsheet, or other document with this information.	Not automatically documented in the EHR.<br><br>Must be updated regularly to keep up to date.	.SDHHANDOUTS can document services for which you gave the patient information.<br><br>Make list maintenance the responsibility of the staff person who updates preference lists.
2. Create and maintain preference lists in your EHR for SDH referrals. List local agencies to which your teams often refer patients.	Staff may already know how to use preference lists.<br><br>EHR-based; enables tracking.	Must be updated regularly.	Make list maintenance the responsibility of the staff person who updates preference lists.<br><br>Be sure that the person tasked with using the preference list has security clearance to do so.
3. Don't create your own resource list. Instead, contract with an organization that provides these lists.<br><br>*See below.*	The list is updated for you, so you can keep your binder or preference list (options 1-2) up to date.	These lists are often not free. They are also not comprehensive in all regions.<br><br>A staff person must enter this resource information into a preference list.	Look into the costs and local coverage for the organizations listed below; consider whether they make sense for your clinic.<br><br>Epic 2018 (which will go live for OCHIN members in March 2019) has an option to 'plug in' community resource lists.

How to obtain information on community resources to populate your resource lists.

Google/Web Search: Google can provide information on resources in a given city, zip code, or distance from your clinic. You will have to re-run this search regularly to keep your resource list up.

Pro: Google is free. *Con:* It is not always up-to-date.

Social Service Resource Locators (SSRLs): Several companies offer continuously updated directories of community social services: e.g., 211 (http://www.211.org/), Aunt Bertha (https://www.auntbertha.com/), Healthify (https://www.healthify.us/), HealthLeads (https://healthleadsusa.org/), and NowPow (http://www.nowpow.com/). Some considerations for working with these companies include:

- *Platform:* Most SSRLs use web-based applications to provide resource lists. They may also include case management tracking and coordination features.
- *Coverage:* No one SSRL currently has resource directories for every community.
- *Cost:* Most SSRLs charge an ongoing fee to use their service; some may also charge setup fees to help them establish an initial directory for your community.

Another option is the American Association of Family Physicians' Neighborhood Navigator: https://www.aafp.org/patient-care/social-determinants-of-health/everyone-project/neighborhood-navigator.html

GUIDE TO SOCIAL DETERMINANTS OF HEALTH SCREENING AND REFERRAL-MAKING USING THE EHR

THE ASCEND PROJECT

KAISER PERMANENTE®
Center for Health Research

OCHIN

Step 4: Train Clinic Staff in the 'SDH Plan'

Your next step is to let your clinic staff know about your SDH Plan, including intended workflows and how they will be rolled out, and how to use the EHR tools for SDH. This guide will help clinic leaders and the SDH Champion conduct this training. It includes training slides that you can adapt for your clinic.

Key Points for Orienting Clinic Staff to Screening and Making Referrals on SDH, Based on Other CHCs' Experiences 3

Orientation Slide Deck 5

If you need help with any of the aspects of this step, the OCHIN Implementation Support Team can help you. Please contact Julianne Bava at 503-943-5774 or ASCEND@ochin.org. The Implementation Support Team office hours will provide additional help from the OCHIN team and from other CHCs that are implementing SDH screening and referrals. For step-by-step instructions on using the SDH tools in Epic, see ASCEND SDH user guides in Ella.

Step 4: Key Points for Orienting Staff to Screening and Making Referrals on SDH, based on Other CHCs' Experiences

Q: Why collect SDH data if we can't refer patients to resources to address a given SDH need?

- SDH profoundly influence health, so they should be considered in care decisions. Staff at other CHCs report that SDH screening often provides previously unknown information about both new *and* established patients, and can inform care planning.
- SDH data can be used to assess needs in your community. This can help clinic leaders advocate for resources, develop community partnerships, and target investments.
- Some CHCs can use SDH data to adjust payment rates based on their patients' social complexity, or to convince funders to cover non-billable services. Others link SDH data to reporting requirements.
- Try to ensure that your staff understand why SDH data are being collected!

Q: Which SDH needs should we screen for? Do we have to ask the whole SDH questionnaire?

- There is no single or "right" way to do this; *your clinic can choose* which SDH measures you want to screen for. The Step 2 documents walk you through your options.
- ***Remember***: In the EHR, you can use the toggle buttons at the top of the SDH screening flowsheet to expand or hide certain items. You can choose to ask the "Full SDH Questionnaire" (a.k.a., *PRAPARE*), or the "AHC Questionnaire," or just individual SDH measures, depending on *your* choices. (If you're using the paper version of the OCHIN SDH questionnaire available on Ella, you can also edit it to include only the questions you want to ask).

Q: Do we need to ask the questions exactly as written?

- No. It is OK to customize the question wording if that seems appropriate for a given patient, or to weave the questions into a more general conversation.

Q: How can staff avoid upsetting patients when we ask these potentially sensitive questions?

- Other CHCs report that patients are rarely upset by the SDH questions. In fact, they often appreciate being asked.
- Administer the questionnaire in a private area, if possible.
- Let the patient know that screening is universal, not targeted at them (*e.g.,* "We are asking all new patients these questions").
- Be clear about why this information is being collected (e.g., "So we can better help you"), and how it will be used.

Q: How might SDH screening affect staff, or staff relationships with their patients?

- Some CHCs say that the SDH questionnaire can open the door to in-depth discussions with the patient about their needs. This can help staff feel engaged in patient care, and support patient-centered care. (However, some staff may be upset by the amount of reported need, or if they cannot provide immediate help).
- Sometimes hearing about SDH-related challenges can be upsetting to staff. Remind those conducting the screening to take care of themselves, and give them space to rest, take a break, or access counseling as needed.

    - **TIP:** See Oregon Primary Care Association: https://www.orpca.org/files/8%20Principles%20for%20Patient-Centered%20Social%20Determinants%20of%20Health%20Screening.pdf

    - **TIP:** Conduct SDH screening in a way that supports relationship-building.
    - **TIP:** The question on stress can be a good ice-breaker.

Step 4: Key Points for Orienting Staff to Screening and Making Referrals on SDH, based on Other CHCs' Experiences

Q: What do clinic staff need to know to support SDH screening / referral adoption?

- Ensure that staff are comfortable introducing the subject and asking the SDH questions.
- Ensure that staff know how to enter the SDH in the Epic flowsheets.
- Ensure that staff know how to follow up on positive SDH screening results - e.g., how to: acknowledge need, hand the patient off to a staff person who can help, make internal or external referrals, and / or give information about community resources.

- **TIP:** Consider role-playing to increase staff skills and confidence.

Keep in mind: The Oregon Primary Care Association has a set of guiding principles, termed Empathic Inquiry, that synthesizes motivational interviewing and trauma-informed care and applies them to SDH screening. See: https://www.orpca.org/files/8%20Principles%20for%20Patient-Centered%20Social%20Determinants%20of%20Health%20Screening.pdf

Orientation Slide Deck

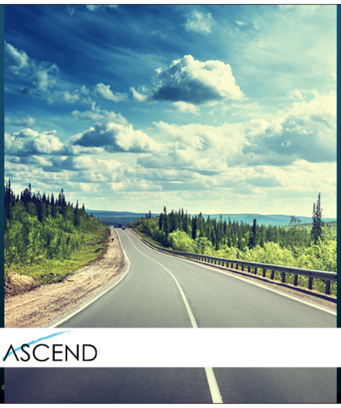

1

Social Determinants of Health Screening Kick-Off / Orientation

Using OCHIN Epic SDH Tools (including PRAPARE) for SDH Data Collection, Screening, and Referral

Clinic Name

Date

ASCEND

WE ARE OCHIN

What are Social Determinants of Health (SDH)?

2

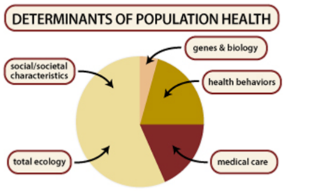

Tarlov, A.R., Public Policy Frameworks for Improving Population Health. Annals of the New York Academy of Sciences, 1999. 896(SOCIOECONOMIC STATUS AND HEALTH IN INDUSTRIAL NATIONS: SOCIAL, PSYCHOLOGICAL, AND BIOLOGICAL PATHWAYS): p. 281-293.

- SDH are the conditions in which people live and work. They profoundly impact health risks and outcomes, and ability to act on care recommendations.
- Only 10-20% of health outcomes are attributed to clinical care; **SDH account for 60-80% of health outcomes.**
- SDH that impact health include: Housing stability; food security; access to transportation and childcare; ability to pay for basic utilities, etc.

© 2016 OCHIN. This material contains confidential and copyrighted information of Epic Systems Corporation.

WE ARE OCHIN

Social Determinants of Health (SDH)

3

SDH that you can document in the OCHIN Epic EHR include:

- *Household income*
- *Education*
- *Housing status*
- *Food security*
- *Social connection / isolation*

© 2016 OCHIN. This material contains confidential and copyrighted information of Epic Systems Corporation.

WE ARE OCHIN

Why Collect SDH?

4

- Understand the factors affecting your patients' health
- Adapt treatment and care planning as needed
- Identify needed referrals to community social services
- Enable targeted outreach
- Demonstrate areas of need for resourcing and advocacy

To walk through reasons for collecting SDH and see how it can affect your clinic's activities, see "Identifying Your Organization's Goals for SDH Documentation" part 1

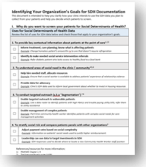

© 2016 OCHIN. This material contains confidential and copyrighted information of Epic Systems Corporation.

WE ARE OCHIN

Clinic Goals

5

- Which patients does your clinic want to screen?
- Which SDH do you want to screen for?
- How often do you want to screen patients?
- How / when in workflows will you collect SDH data? Who will collect it?
- Will your clinic use SDH data for panel management or outreach efforts?

See "Identifying Your Organization's Goals for SDH Documentation" parts 2-4

© 2016 OCHIN. This material contains confidential and copyrighted information of Epic Systems Corporation.

WE ARE OCHIN

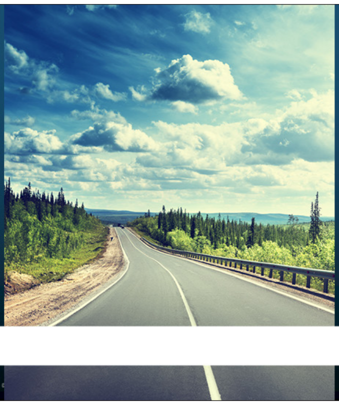

6

EHR Tools To Document and Address Patients' Social Determinants of Health

WE ARE OCHIN

Our EHR's SDH Tools Can Be Used To…

7

- **Identify** the patients who are targeted for SDH screening
  - At check-in / rooming
- **Review** SDH screening rates among targeted patients
- **Document** SDH at different workflow steps
  - Before the visit
  - During a clinic visit or outreach encounter
- **Refer** patients with SDH needs to social services and **track** past referrals
- **Review** a given patient's SDH needs

© 2016 OCHIN. This material contains confidential and copyrighted information of Epic Systems Corporation.

WE ARE OCHIN

Our EHR's SDH Tools Can Be Used To…

8

- **Identify** the patients who are targeted for SDH screening
  - At check-in / rooming
- **Review** SDH screening rates among targeted patients
- **Document** SDH at different workflow steps
  - Before the visit
  - During a clinic visit or outreach encounter
- **Refer** patients with SDH needs to social services and **track** past referrals
- **Review** a given patient's SDH needs

© 2016 OCHIN. This material contains confidential and copyrighted information of Epic Systems Corporation.

WE ARE OCHIN

Orientation Slide Deck

You Can *Identify* Patients You Want to Screen for SDH in Reporting Workbench

9

- Create a Reporting Workbench roster that lists targeted patients (OCHIN team will help set this up)
- Assign an SDH Questionnaire series to those patients

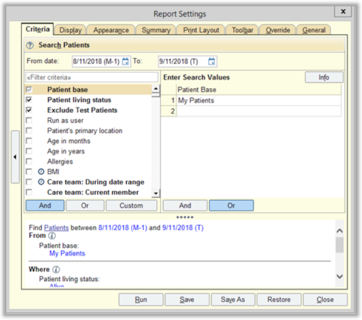

© 2016 OCHIN. This material contains confidential and copyrighted information of Epic Systems Corporation.

Once the Questionnaire Series is Assigned, You Can…

10

- Send patients a **letter**, **email**, or **text** asking them to complete SDH screening in the patient portal (including signing up for the portal)
- **Identify** assigned patients at **check-in** (DAR)
- **Identify** assigned patients at **rooming** (Provider Schedule / Snapshot)

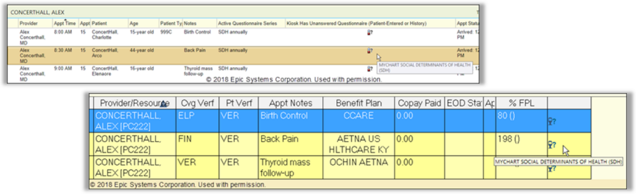

© 2016 OCHIN. This material contains confidential and copyrighted information of Epic Systems Corporation.

Once the Questionnaire Series is Assigned, You Can…

11

- Review how many targeted patients you have screened
- Sort them as needed
- Conduct outreach as needed

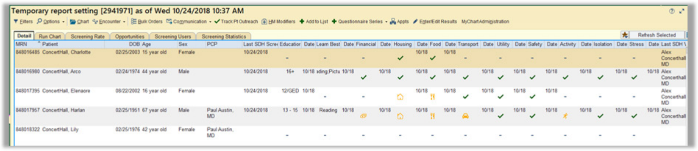

© 2016 OCHIN. This material contains confidential and copyrighted information of Epic Systems Corporation.

You Can *Document* SDH Screening Before the Visit…

12

- Use the Reporting Workbench roster described above to contact scheduled patients and ask them to complete the SDH screening in the portal
- Data entered through the portal is sent to the EHR!

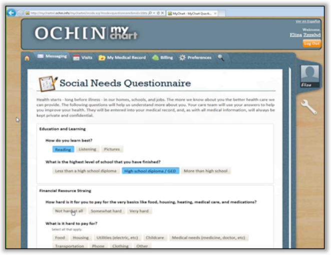

© 2016 OCHIN. This material contains confidential and copyrighted information of Epic Systems Corporation.

You Can *Document* SDH Screening at the Visit…

13

- In **Screening** or **Flowsheets**
- These options let you **choose** which SDH screening you want to use:
  - PRAPARE
  - AHC
  - Individual SDH domains
- You can also **document**:
  - Whether the patient **wants** clinic support addressing a given SDH need
  - **What kind** of support the patient wants
  - **Which SDH** the patient wants help with

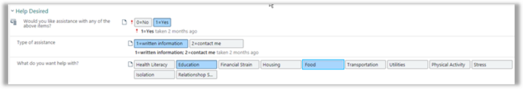

© 2016 OCHIN. This material contains confidential and copyrighted information of Epic Systems Corporation.

AHC Clinics Can Submit SDH Data Through the EHR!

14

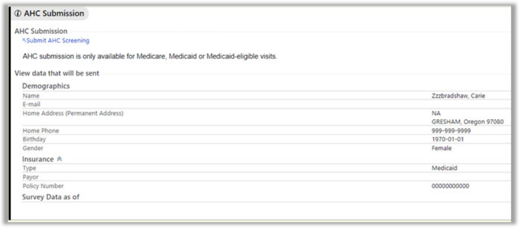

© 2016 OCHIN. This material contains confidential and copyrighted information of Epic Systems Corporation.

Orientation Slide Deck

Our EHR's SDH Tools Can Be Used To... 15

- **Identify** the patients who are targeted for SDH screening
  - At check-in / rooming
- **Review** SDH screening rates among targeted patients
- **Document** SDH at different workflow steps
  - Before the visit
  - During a clinic visit or outreach encounter
- **Refer** patients with SDH needs to social services and **track** past referrals
- **Review** a given patient's SDH needs

© 2016 OCHIN. This material contains confidential and copyrighted information of Epic Systems Corporation.

WE ARE OCHIN

Our EHR's SDH Tools Can Be Used To... 16

- **Identify** the patients who are targeted for SDH screening
  - At check-in / rooming
- **Review** SDH screening rates among targeted patients
- **Document** SDH at different workflow steps
  - Before the visit
  - During a clinic visit or outreach encounter
- **Refer** patients with SDH needs to social services and **track** past referrals
- **Review** a given patient's SDH needs

© 2016 OCHIN. This material contains confidential and copyrighted information of Epic Systems Corporation.

WE ARE OCHIN

You Can Create a Social Service *Referral Preference List* and... 17

- **Order** referrals from this list
- These 'orders' will appear in the **AVS**
- And they will be **documented** in the chart for later review

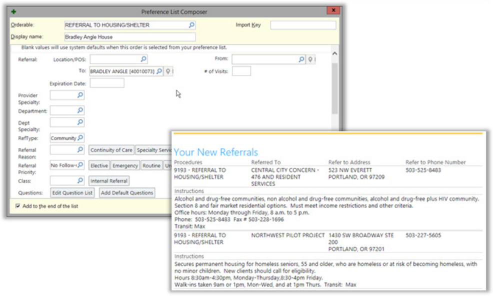

© 2016 OCHIN. This material contains confidential and copyrighted information of Epic Systems Corporation.

WE ARE OCHIN

You Can Also *Code* SDH Information Into... 18

- Problem List
- Visit diagnoses
- We will give you a list of codes!

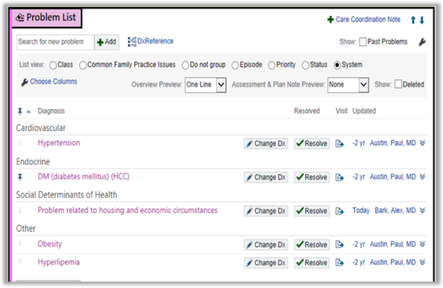

© 2016 OCHIN. This material contains confidential and copyrighted information of Epic Systems Corporation.

WE ARE OCHIN

You Can *Review* SDH Needs... 19

- In the patient header

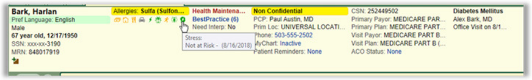

- In Snapshot

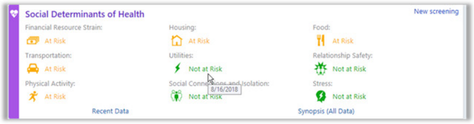

© 2016 OCHIN. This material contains confidential and copyrighted information of Epic Systems Corporation.

WE ARE OCHIN

You Can *Review* SDH Needs... 20

- In the Social Determinants Report
- In Screening

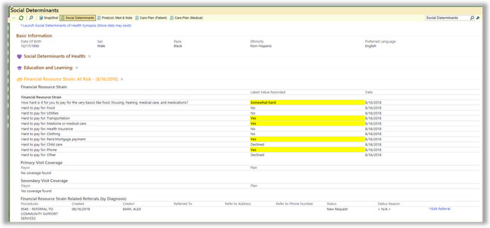

© 2016 OCHIN. This material contains confidential and copyrighted information of Epic Systems Corporation.

WE ARE OCHIN

Some *Tips* for Documenting SDH Referrals…

21

- You can **document SDH referrals** in Progress Notes with a SmartPhrase
- You can also **document if a patient declined assistance** with SDH-related referrals with SmartPhrases
- You may get a **duplicate orders** warning when you make SDH referrals to more than one social service agency (that's OK!)

© 2016 OCHIN. This material contains confidential and copyrighted information of Epic Systems Corporation.
WE ARE OCHIN

Our EHR's SDH Tools Can Be Used To…

22

- **Identify** the patients who are targeted for SDH screening
  - At check-in / rooming
- **Review** SDH screening rates among targeted patients
- **Document** SDH at different workflow steps
  - Before the visit
  - During a clinic visit or outreach encounter
- **Refer** patients with SDH needs to social services and **track** past referrals
- **Review** a given patient's SDH needs

© 2016 OCHIN. This material contains confidential and copyrighted information of Epic Systems Corporation.
WE ARE OCHIN

DISCUSSION

23

What are potential barriers to adopting SDH data collection at our clinic?

Examples:

- *Lack of staff time*
- *Concerns about asking sensitive SDH-related questions*
- *Limited ability to act on patients' identified social needs*

© 2016 OCHIN. This material contains confidential and copyrighted information of Epic Systems Corporation.
WE ARE OCHIN

You Can *Review* SDH Needs…

24

- In Synopsis (includes SDH data over time!)

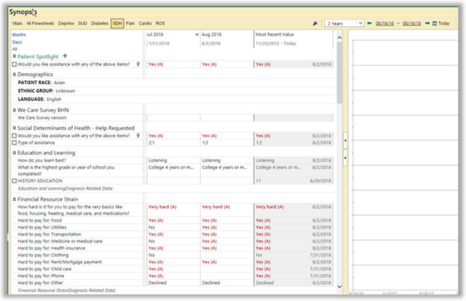

© 2016 OCHIN. This material contains confidential and copyrighted information of Epic Systems Corporation.
WE ARE OCHIN

Detailed Instructions for Using These EHR Tools Are in ELLA

25

Go to: ella.ochin.org

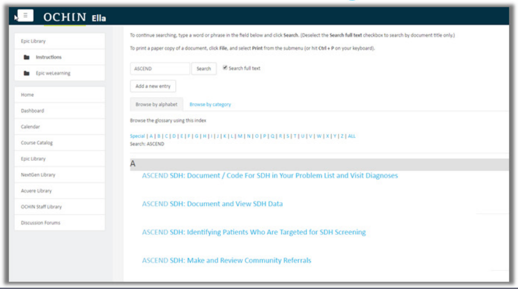

© 2016 OCHIN. This material contains confidential and copyrighted information of Epic Systems Corporation.
WE ARE OCHIN

26

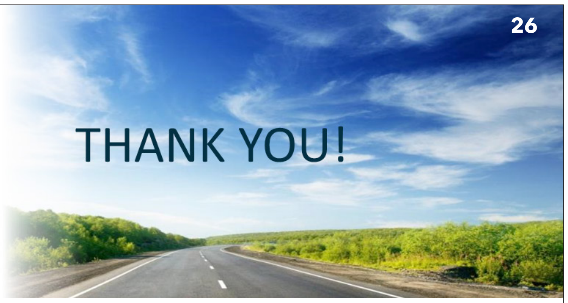

Contact the ASCEND team at:
bavaj@ochin.org
ascend@ochin.org

WE ARE OCHIN

GUIDE TO SOCIAL DETERMINANTS OF HEALTH SCREENING AND REFERRAL-MAKING USING THE EHR

THE ASCEND PROJECT

KAISER PERMANENTE®
Center for Health Research

OCHIN

Step 5: Roll Out and Iterate your SDH Plan

Your final step is to implement your SDH plan, and revise it as needed.
This guide will help your SDH Champion in these tasks.

**PDSA Cycle Worksheet – SDH Example**	**2**
**Plan, Do, Study, Act (PDSA) Cycle Checklist**	**5**
**Additional PDSA Tips**	**7**

If you need help with any of the aspects of this step, the OCHIN Implementation Support Team can help you. Please contact Julianne Bava at 503-943-5774 or ASCEND@ochin.org. The Implementation Support Team office hours will provide additional help from the OCHIN team and from other CHCs that are implementing SDH screening and referrals. For step-by-step instructions on using the SDH tools in Epic, see ASCEND SDH user guides in Ella.

PDSA Cycle Worksheet - SDH Example

Plan, Do, Study, Act (PDSA) cycles are a way to test changes in your clinic by planning, doing, observing results, and acting on what is learned. Use this document as a guide to help you develop PDSAs for SDH screening and data collection workflows in your clinic. Improvement and PDSAs are ongoing, as you apply what you learn from each cycle. **Here is an example of a SDH implementation PDSA cycle.**

**PDSA Cycle Name and Brief Description**<br>Test time required to screen and document SDH.<br>Cycle description: data collection for documentation time of SDH screening	**Start Date:**<br>9/1/18	**End Date:**<br>9/8/18
**Objective of Cycle**:<br>Test efficiency of MA SDH screening and documentation	**Cycle #:**<br>1	**Cycle Owner:**<br>RN Care Manager

PLAN: Identify questions. Predict results. Determine data to be collected and by whom.

Questions	Predictions
1. How long will it take to enter screening results into the workflow?	1. Entering screening results into flow sheet will take 8 minutes.
**Data to be collected**	**Who collects data for how long**
1. Time needed to screen patients with X questions.<br>2. Time needed to enter screening results into flowsheet.	1. MA will keep track of time it takes to ask and enter screening results in Epic.<br>2. MA will track time points for one week.

DO: Carry out the change or activity, collect the data. Document what happened (+/-).

Entering screening results into Epic took an average of 2 minutes per screening.

Step 5: Roll Out and Iterate your SDH Plan

**STUDY**: Summarize what you learned; identify any new questions / issues; compare with predictions; compare results across teams / MAs.
**Learnings:** Entering screening results into SDH flowsheet did not take as long as predicted. We also learned that some patients did not want assistance, despite reported SDH difficulties.
**Results:** No significant time burden to enter SDH screening results into Epic flowsheet.
**New Issues or Questions:** We need to track patients who decline assistance, yet identify difficulties, so that someone can check-in at their next visit. We need to update our list of external resources.
**ACT**: Determine next steps based on what you learned in this cycle. Choose whether to test under different conditions (e.g. different day of the week) to confirm or disprove improvements.
Continue entering SDH screening results into Epic. Update our list of external resources.
**Next Cycle:** Track the number of patients identifying SDH needs yet declining assistance.
**Ad Hoc Members:** CHWs, BH providers, MAs

Plan, Do, Study, Act (PDSA) Cycle Checklist

	Steps	Considerations
**Plan**	• Define cycle's **Objective Type**: Collect Data/Develop Change; Test a Change; Implement a Change<br>• Define specific **Questions** to be answered from this cycle<br>• When possible, make a **Prediction(s)** about the answer to each question, and note the basis for the prediction<br>• Define the **Action Plan** to answer the questions: What (actions), Who, Where, When, How<br>• Create a detailed plan for **Data Collection and Analysis**	• Are data available to answer the questions, or will new data be required?<br>• Does the team agree on some / all of the predictions?<br>• What tools and methods will be used to collect and analyze the data?<br>• Did you assign responsibilities for collection and analysis of the data?<br>• Is training needed for individuals collecting data?<br>• Is the plan consistent with the project charter?<br>• Can the plan be tested on a small scale?<br>• Have you considered how people outside the team will be impacted by this plan?<br>• Has a change management plan been considered?
**Do**	• Carry out the plan<br>• Capture observations in carrying out the plan, especially if unexpected<br>• Begin analysis of data	• What (if anything) happened that you did not expect / anticipate?<br>• Did anything go wrong?<br>• Were there any 'special causes' / events that affected the data you collected?
**Study**	• **Analyze** the data and observations<br>• Compare data with **predictions**<br>• Summarize **what was learned** (new knowledge) in this cycle<br>• Develop / update graphical tools; include (link or copy) in PDSA cycle document	• Do results of the cycle agree with predictions made in the planning phase?<br>• Under what conditions could the conclusions from this cycle be different?<br>• What are the implications of the unplanned observations / problems in the Do Step?<br>• Do the data and observations help answer the questions posed in the plan?<br>• Are charts, graphs or diagrams annotated with what was changed / learned?<br>• Can learning be applied in other areas?

**Act**	• List any changes that can be made to the process<br>• Define the **objective type** and **objective** for the next PDSA cycle	• What is the next cycle objective, based on learning from this cycle?<br>• Are you ready to develop possible changes? What are your theories?<br>• Are we ready to test a change?<br>• Are we ready to implement a change?<br>• Do we still need to collect data in order to understand the current situation?

Additional PDSA Tips

- To get input from various points in the process that is valuable to improvements/learnings, always complete the Study Step with all members involved in SDH collection
- Assign a PDSA Cycle Lead to coordinate the cycle
- Consider completing PDSA to coincide with team meetings (e.g., team meetings occur every Monday, run a PDSA cycle from Monday to Monday)
- Move to testing changes when you have good theories and changes to test
- Test on a small scale to maximize learning and reduce risk
- Implementation plans should consider a change management plan- this will ensure control in schedules, scope, communication, and resources to minimize the impact of a change on staff, patients, and the clinic
- Document only enough to ensure there is a well-defined plan, observations and data are collected and analyzed, and learning is captured
- The Act step becomes the beginning of the Plan step on the next cycle

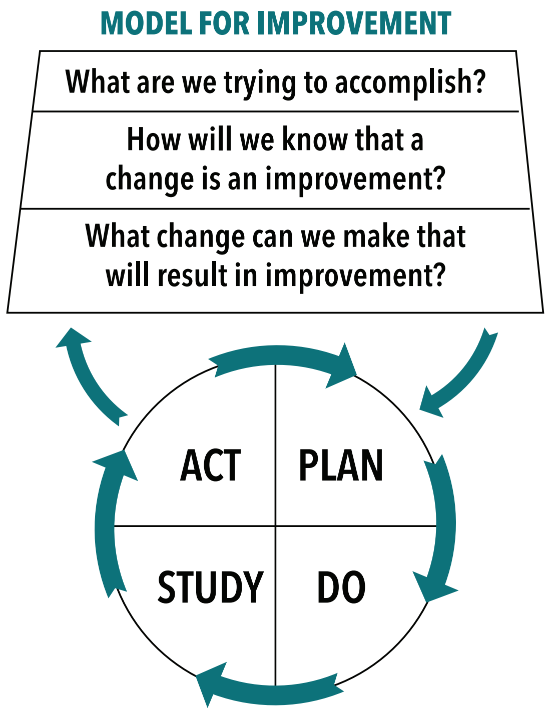

Supplement: Supplementary file 1 — Guide to social determinants of health screening and referral-making using the electronic health record. (PDF 4124 kb) [file 13012_2019_855_MOESM1_ESM.pdf]
