# Supplementary material for: Study protocol: a pragmatic, stepped-wedge trial of tailored support for implementing social determinants of health documentation/action in community health centers, with realist evaluation
Source: Implement Sci. 2019 Jan 28;14:9. doi: 10.1186/s13012-019-0855-9 (PMC6348649; doi:10.1186/s13012-019-0855-9)
Supplement: Supplementary file 2 — Baseline survey. (DOCX 23 kb) [file 13012_2019_855_MOESM2_ESM.docx]

**Additional file 2: Baseline Survey**

*Please complete the following survey, which is designed to collect information about your clinic for the ASCEND study. We suggest that your SDH Project Champion complete this survey, with input from others in your practice (e.g., Medical Director, Billing Manager) as needed.*

| **Clinic: __________________________________________________________**  **Date: ___________________________________________________________**  **Name of Person entering data: _____________________________________________**  **Clinic role of person competing this form: ____________________________** | | | | |
| --- | --- | --- | --- | --- |
|  | | | | |
|  |  | ***If Y, please describe*** | | |
| 1. Have any major disruptive events, e.g., change in clinic leadership, funding, or turnover of key staff, occurred at your clinic in the last 6-12 months? | **Y / N / Don’t Know** |  | | |
| 2. Are patients at your clinic assigned to specific practice panels? | **Y / N / Don’t Know** |  | | |
| 3. Are any other team-based strategies in place at your clinic? | **Y / N / Don’t Know** |  | | |
| 4. Is your clinic involved in any other initiatives related to SDH? | **Y / N / Don’t Know** |  | | |
| 5. Are there any *external* policies / incentives that might impact adoption of the clinic’s SDH Plan? | **Y / N / Don’t Know** |  | | |
| 6. Are there any *internal* policies / incentives that might impact adoption of the clinic’s SDH Plan? | **Y / N / Don’t Know** |  | | |
| 7. Are there currently staff at your clinic who help patients with non-clinical needs (e.g., CHW, social worker, behaviorist)? | **Y / N / Don’t Know** |  | | |
| 8. Is there someone at your clinic who knows how to run reports from Reporting Workbench? | **Y / N / Don’t Know** |  | | |
| 9) What is the ownership structure of this clinic? [check all that apply] | **( ) Clinician owners**  **( ) Hospital / hospital system / health system**  **( ) University / university system**  **( ) HMO or other insurance entity**  **( ) Government (federal / state / local)**  **( ) Not-for-profit corporation**  **( ) Other (specify)** |  | | |
| 10) What is the most common type of provider payment structure in your clinic? [check all that apply] | **( ) Salaried**  **( ) Fee for service**  **( ) Mix of salaried and fee for service**  **() Other (specify)** |  | | |
|  | | | | |
|  | ***Select the answer that is most accurate for your clinic.*** | | | |
| 8a. In general, non-physician practice team members … | …play a limited role in providing clinical care. | …are primarily tasked with managing patient flow and triage. | …provide some clinical services, e.g., assessment or self-management support. | …perform key clinical service roles that match their abilities and credentials. |
| 8b. In general, providers (Physicians, NP/PAs) and clinical support staff ... | …work in different pairings every day. | …are arranged in teams but are frequently reassigned. | …consistently work with a small group of providers or clinical support staff in a team. | …consistently work with the same provider/clinical support staff person almost every day. |
| 8c. In general, workflows for clinical teams ... | …are not documented and/or are different for each person or team. | …are documented,  but not used to  standardize workflows across the practice. | … are documented  and utilized to  standardize practice. | …are documented,  utilized to standardize workflows, and are regularly evaluated and modified. |
| 8d. In general, standing orders that can be acted on by non-physicians under protocol ... | …do not exist for the  practice. | …are developed for  some conditions but are not regularly used. | …are developed for some conditions and are regularly used. | …are developed for many conditions and are used extensively. |
| 9. In general, population level* health outcomes ...  ** “Population-level” means measured as a percentage or rate within a specific group or panel of patients.* | …are not measured. | …are measured but not tracked to see changes over time. | …are measured and tracked. | …are measured and tracked, with regular reviews and efforts to improve care delivery and outcomes. |
| 9a. If these rates ARE measured … | - | By whom? _______________ | By whom? _______________ | By whom? _______________ |
|  | | | | |
|  | ***Please describe.*** | | | |
| 12. AT COMPLETION OF WEDGE ONLY:  Have there been any unintended / unanticipated effects of deciding to implement / implementing SDH screening? |  | | | |
